# High yield production of 1,4-cyclohexanediol and 1,4-cyclohexanediamine from high molecular-weight lignin oil†

**DOI:** 10.1039/d2gc03777g

**Published:** 2022-11-29

**Authors:** Xianyuan Wu, Mario De bruyn, Julia Michaela Hulan, Henrique Brasil, Zhuohua Sun, Katalin Barta

**Affiliations:** Stratingh Institute for Chemistry, University of Groningen Groningen The Netherlands; Beijing Key Laboratory of Lignocellulosic Chemistry, Beijing Forestry University No. 35 Tsinghua East Road Haidian District Beijing 100083 P. R. China; Department of Chemistry, Organic and Bioorganic Chemistry, University of Graz Heinrichstrasse 28/II 8010 Graz Austria katalin.barta@uni-graz.at

## Abstract

The complete utilization of all lignin depolymerization streams obtained from the reductive catalytic fractionation (RCF) of woody biomass into high-value-added compounds is a timely and challenging objective. Here, we present a catalytic methodology to transform beech lignin-derived dimers and oligomers (DO) into well-defined 1,4-cyclohexanediol and 1,4-cyclohexanediamine. The latter two compounds have vast industrial relevance as monomers for polymer synthesis as well as pharmaceutical building blocks. The proposed two-step catalytic sequence involves the use of the commercially available RANEY® Ni catalyst. Therefore, the first step involves the efficient defunctionalization of lignin-derived 2,6-dimethoxybenzoquinone (DMBQ) into 1,4-cyclohexanediol (14CHDO) in 86.5% molar yield, representing a 10.7 wt% yield calculated on a DO weight basis. The second step concerns the highly selective amination of 1,4-cyclohexanediol with ammonia to give 1,4-cyclohexanediamine (14CHDA) in near quantitative yield. The ability to use RANEY® Ni and ammonia in this process holds great potential for future industrial synthesis of 1,4-cyclohexanediamine from renewable resources.

## Abstract 2

we present a highly efficient catalytic strategies which are capable of converting DMBQ obtainable from high molecular-weight lignin-oil into industrially highly relevant and well-defined 1,4-cyclohexanediol and 1,4-cyclohexanediamine.

## Graphical abstract

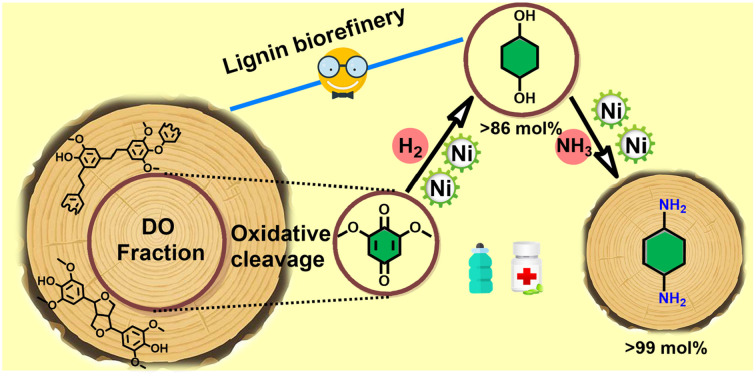

## Introduction

Currently, a high demand exists for the development of sustainable catalytic methodologies for known commodity chemicals from renewable resources such as lignocellulosic biomass.^1–11^ In this respect, reductive catalytic fractionation (RCF) is a powerful tool to catalytically fractionate lignocellulosic biomass.^12–15^ Typically, this results in the depolymerization of lignin into monomeric and oligomeric fractions while largely retaining the structural integrity of the cellulose component. Recent research attention is given to the valorization of the oligomeric lignin fraction as its further deconstruction would greatly enhance the overall achievable monomeric yield. In this respect, the group of Samec recently developed an elegant oxidative process to convert part of the oligomeric lignin fraction featuring distinct C–C linkages obtained upon RCF to 2,6-dimethoxybenzoquinone (DMBQ),^16^ a compound with wide synthetic applications.^17^

**Fig. 1:**
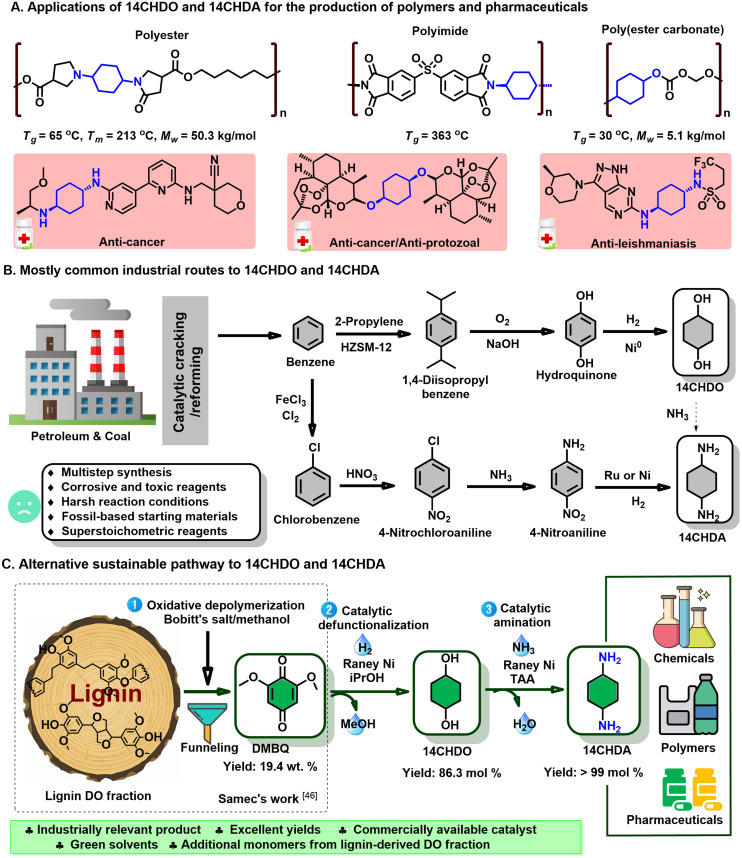
Existing and potential future strategies to produce 14CHDO and 14CHDA. (A) Applications of 14CHDO and 14CHDA for the production of polymers and pharmaceuticals; (B) Traditional industrial and prospective pathways for the production of 14CHDO and 14CHDA, with the central starting compound being fossil-derived benzene. More details can be found in ESI Note 1.† (C) Here proposed and developed three-step catalytic pathway to 14CHDO and 14CHDA from lignocellulosic biomass: (1) Oxidative cleavage of the RCF oligomeric lignin fraction to DMBQ using Bobbitt's salt.^16^ (2) Demethoxylation and hydrogenation of DMBQ to 14CHDO over RANEY® Ni catalyst. (3) Catalytic amination of 14CHDO to 14CHDA over RANEY® Ni catalyst.

Here, we present the development of feasible catalytic strategies to convert DMBQ into industrially highly relevant and well-defined 1,4-cyclohexanediol (14CHDO) and 1,4-cyclohexanediamine (14CHDA). 14CHDO is a valuable compound that has been shown to be useful as a monomer to produce polycarbonates, polyethers and polyesters including scratch-resistant coatings as well as supramolecular associations owing to the distinct *cis*/*trans* isomerism of this molecule (Fig. 1A).^18–21^ 14CHDO serves as a direct synthetic precursor to a range of pharmaceuticals, typically by the functionalization of one or both hydroxyl groups.^22^ Exemplary in this respect are the synthetic routes to phenyl cyclohexylcarboxamides, dihydroartemisinin and derivatives, and compounds with distinct anti-cancer properties.^22^ 14CHDO is also the prime industrial precursor to 1,4-cyclohexanedione,^23^ a highly versatile platform molecule for the production of pharmaceuticals such as the analgesic Cebranopadol.^24,25^ The prime applications of diamines such as 14CHDA are in the preparation of polyamides,^26,27^ polyimides,^28^ polyureas,^29^ polyurethanes^30^ and biologically active compounds (Fig. 1A).^31,32^

Industrially, 14CHDO and 14CHDA are produced from fossil resources, relying on the use of benzene, a carcinogenic and mutagenic compound with a lengthy hazard classification, by several strategies that are briefly summarized in Fig. 1B and explained in ESI Note 1† in more detail.^33^ Central to the synthesis of 14CHDO is the formation of hydroquinone (HQ),^34^ while 14CHDA can be prepared by hydrogenation of 1,4-phenylenediamine (14PDA).^35,36^ However, these processes have specific drawbacks such as multi-step synthesis routes and the use of super stoichiometric reagents under harsh reaction conditions.

In this work, we present a catalytic alternative (Fig. 1C) that starts from lignin-derived depolymerization streams and relies on a two-step RANEY® Ni based sequence capable of converting DMBQ into 14CHDA. More specifically, the first step comprises the reductive demethoxylation and hydrogenation of DMBQ into 14CHDO in up to 86.5% yield, while the second step features a near-quantitative amination of 14CHDO into 14CHDA in the presence of ammonia. The latter transformation fits the quest for sustainable catalytic methodologies to introduce nitrogen in biomass-derived chemicals.^37–49^ To illustrate the robustness of this method, we also show that 10.7 wt% 14CHDO (based on a DO fraction basis) can be derived from a real RCF oligomeric lignin fraction.

## Results and discussion

### Catalytic demethoxylation and hydrogenation of DMBQ to 14CHDO

**Fig. 2:**
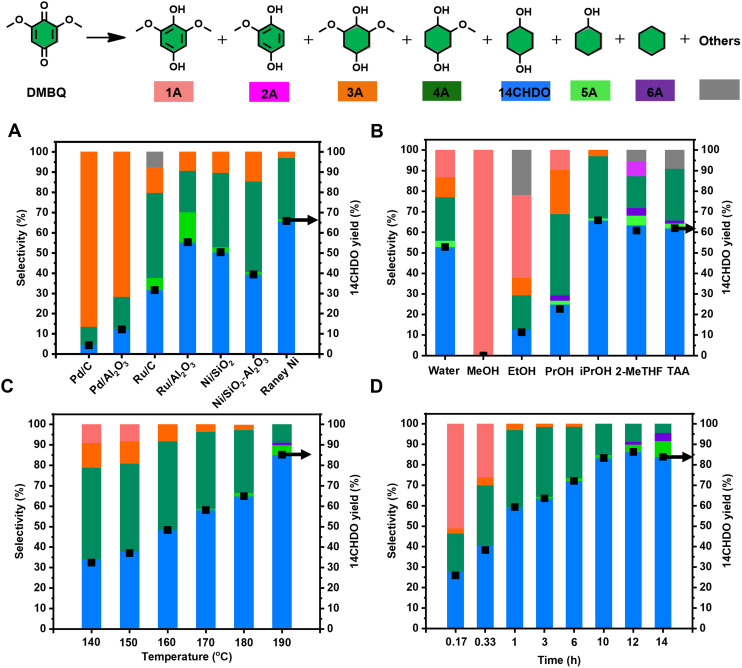
Establishment of the optimal reaction conditions for the catalytic conversion of DMBQ to 14CHDO (A–D); Unless otherwise specified, standard reaction conditions are used: DMBQ (1 mmol, 0.200 g), 200 mg RANEY® Ni, 170 °C, 4 h, 15 mL solvent, 10 mg dodecane. Screening of (A) commercially available heterogeneous metal catalysts. The metal loading of the noble metal catalyst is 5 wt%, while it is 65 wt% for Ni/SiO_2_ and Ni/SiO_2_–Al_2_O_3_ and 89 wt% for RANEY® Ni; (B) solvent influence; (C) reaction temperature, 2 h; (D) different reaction time; (A–D). The numerical values are given in the ESI and more specifically Tables S1–S4.† Conversion and yield values were determined by GC-FID using calibration curves and an internal standard; The black square symbol refers to the yield of 14CHDO.

Firstly, the demethoxylation/hydrogenation of DMBQ to 14CHDO was tested using a range of commercially available heterogeneous noble/non-noble catalysts (Fig. 2A and Table S1†) under standard reaction conditions: DMBQ, RANEY® Ni, 170 °C, 4 h, iPrOH, 10 mg dodecane. It was found that Pd/C and Pd/Al_2_O_3_ engaged predominantly in the hydrogenation of the aromatic ring, producing a high yield of the compound 2,6-dimethoxycyclohexane-1,4-diol (3A). This preferred catalytic hydrogenation of the aromatic ring has been demonstrated earlier.^50,51^ While these two catalysts do display some reductive demethoxylation capability, giving 2-methoxycyclohexane-1,4-diol (4A) in the yields of 9.2 and 16.2%, respectively, their effective selectivity to 14CHDO remained nonetheless low, 4.4 and 12.2%, respectively. Changing the active metal from Pd to Ru led to a sizeable increase in the 4A and 14CHDO selectivities. The best results were obtained with Ru/Al_2_O_3_ as the catalyst, showing a 14CHDO selectivity of 55.3%. However, under the given reaction conditions, the Ru/Al_2_O_3_ catalyst also engaged in hydrodeoxygenation, leading to the formation of cyclohexanol (5A) in 15% yield. Additionally, three heterogeneous Ni-containing catalysts were tested, namely Ni/SiO_2_, Ni/SiO_2_–Al_2_O_3_ and RANEY® Ni. From Fig. 2A and Table S1,† it can be inferred that Ni/SiO_2_ delivers a good, 50.4% 14CHDO yield, higher than that obtainable with Ni/SiO_2_–Al_2_O_3_ (39.4%). Gratifyingly, in the presence of RANEY® Ni, the 14CHDO yield increased to 65.9%. The higher catalytic activity of RANEY® nickel was attributed to its excellent hydrogen transfer activity when using isopropanol as the H-donor solvent.^52^ It is noteworthy that none of the tested Ni catalysts engaged in hydrodeoxygenation, with only low levels of 14CHDO being detected. These results are in line with the work published by the Rinaldi group.^52^ It is further noteworthy that our RANEY® Ni process yields *cis*/*trans* 14CHDO in a 40/60 ratio (for ^1^H NMR results, see ESI section 2.4†). In contrast, the hydrogenation of hydroquinone to 14CHDO leads to a 3 : 1 excess of the *cis vs. trans* isomer over a Ni-Sr/γ-Al_2_O_3_ catalyst.^53^

Having established the superiority of the RANEY® Ni catalyst, the influence of the solvent was investigated next (Fig. 2B and Table S2†). Using MeOH as the solvent showed near-exclusive 2,6-dimethoxybenzene-1,4-diol (1A) formation (*i.e.*, DMBQ aromatization), while usage of EtOH gave a blend of 1A, 3A, 4A, and 14CHDO, thus pointing at a somewhat more developed demethoxylation/hydrogenation capability. The limited demethoxylation and hydrogenation capability of RANEY® Ni in the presence of MeOH or EtOH likely relates to the strong adsorption of methyl- and ethyl-alcoholates on the surface of the RANEY® Ni catalyst.^52,54,55^ Interestingly, increasingly better 14CHDO selectivity was obtained when increasing the hydrophobicity and bulkiness of the alcohol solvent. Indeed, in going from propanol to isopropanol, the 14CHDO selectivity increased from 24.9 to 65.9%, while still forming sizeable amounts of 4A (respectively, 39.4% and 30.2%). Isopropanol has shown to be a good partner with RANEY® nickel in the demethoxylation and hydrogenation of aromatic rings compared to other simpler alcohols (methanol, ethanol *etc*.).^52^ Further suppression of 4A formation could be achieved when using *t*-amyl alcohol as the solvent with a selectivity of 25.2%, yet no further advance in 14CHDO selectivity (62%) could be observed. Aside from alcoholic solvents, 2-MeTHF and water were tested. From Fig. 2C, it can be inferred that 2-MeTHF gives similar results in terms of 14CHDO selectivity (63.4%) as observed with *t*-amyl alcohol, but both selectivity values were slightly lower than those (65.9%) in isopropanol. In the presence of water, the observed 14CHDO selectivity falls out lower than that observed with *t*-amyl alcohol and 2-MeTHF, reaching 52.8%.

By adopting isopropanol as the reaction solvent, the influence of the reaction temperature was investigated. Fig. 2C (Table S3†) shows a direct proportionality between the attained 14CHDO selectivity and the reaction temperature with the best 14CHDO selectivity (85.2%) obtained at 190 °C. Advances beyond 190 °C led to a significant formation of 5A and cyclohexane (6A), a set of hydrodeoxygenation products directly derived from the target 14CHDO product. The influence of the reaction time was conveniently assessed at 170 °C. From Fig. 2D (Table S4†), it can be seen that the obtainable 14CHDO selectivity increases with reaction time, and the optimal value centers around the 12–14 h mark with 14CHDO selectivities of 84–86.3%. Applying longer reaction times led to decreased 14CHDO selectivities as the hydrodeoxygenation of 14CHDO to 5A/6A became ever more dominant.

**Fig. 3:**
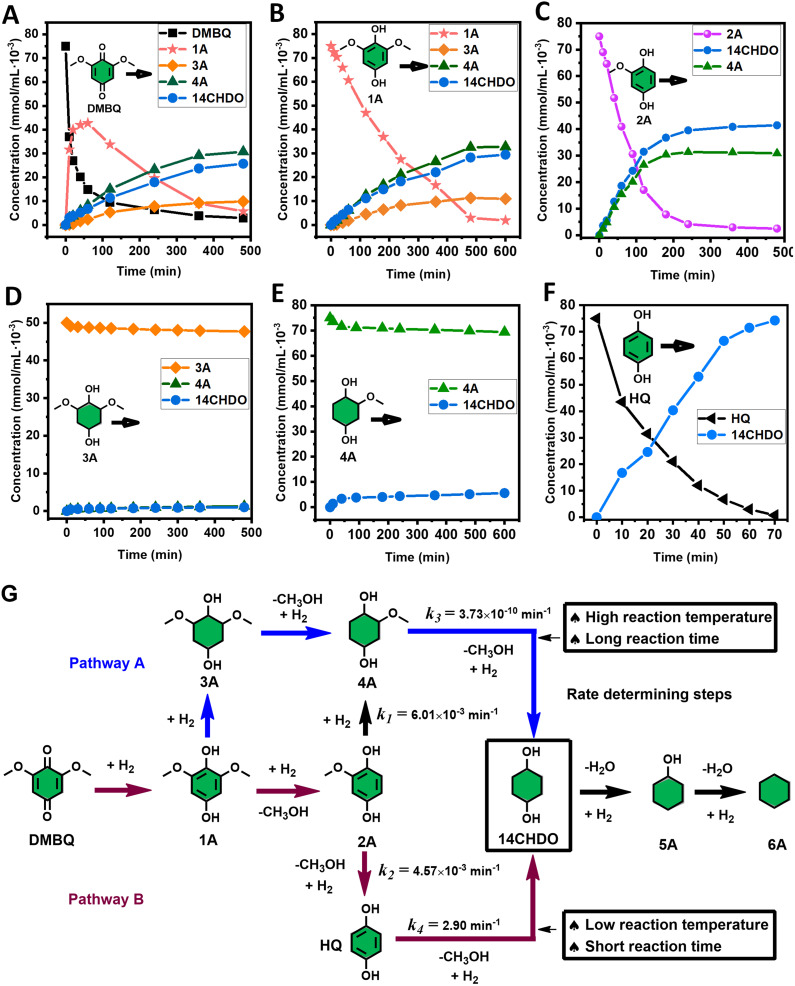
Elucidation of the reaction pathway by means of the reaction kinetics analysis. Reaction conditions: 1.5 mmol substrate, 30 mg RANEY® Ni catalyst, 170 °C, 0–600 min, 20 mL isopropanol, 10 mg dodecane; (A–F) The numerical values for this assessment can be found in the ESI and more specifically in Tables S5–S10.† (D) 1 mmol 3A, 15 mg catalyst (G) proposed reaction network: *k*_1_, *k*_2_, *k*_3_ and *k*_4_ are the apparent kinetic constants obtained from data fitting with a pseudo-homogeneous power-law model.

With the DMBQ-to-14CHDO reaction featuring multiple (potential) simultaneous/consecutive reaction steps, among hydrogenation, demethoxylation and hydrodeoxygenation, we performed a concise study to reveal the main reaction pathway. This is achieved by combining the above gained insights with a series of probe reactions on individual compounds, such as DMBQ, 1A, 2A, 3A, 4A and 14CHDO (Fig. 3A–F, and Tables S5–S10†). In addition, in order to shed light on the reaction mechanism, reaction kinetics were analysed to describe how 2A was transformed into 5A, following a pseudo-homogeneous kinetics model. To simplify the model, the following assumptions were made: (1) the concentration of H_2_ in the liquid phase is constant and the adsorption of H_2_ is the most relevant on the catalyst surface. (2) Compound HQ is rapidly consumed after its production and was treated as an intermediate. (3) The product HQ was not detected in significant amounts. Thus, *k*_4_ ≫ *k*_2_. Based on the assumptions detailed above, the model could be simplified to a power-law model, where *k*_*n*_ are the apparent kinetic constants. The resulting system of ordinary differential equations is the following:
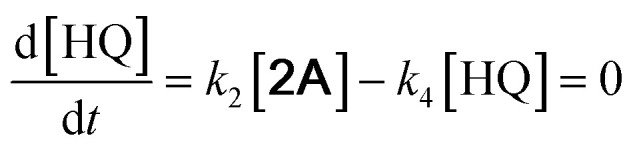

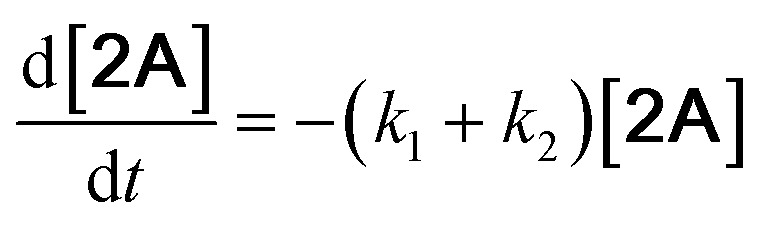

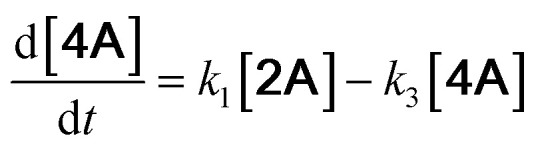

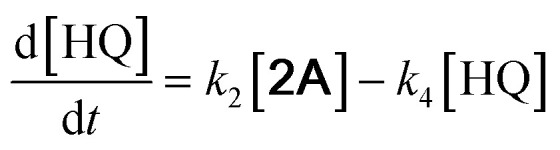

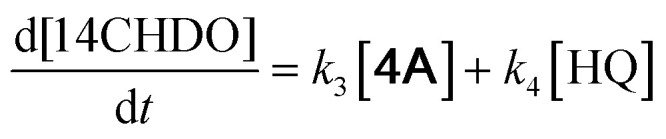


The overall reaction scheme is outlined in Fig. 3G and further discussed below. Firstly, it was found that the DMBQ to 14CHDO reaction occurs chiefly over the 1A intermediate (Fig. 3A). Moreover, it could also be established that the DMBQ to 1A reaction occurs readily in the presence of RANEY® Ni already at RT. Further key findings related to the reaction network were that the rate of the HQ to 14CHDO transformation is orders of magnitude higher than the 4A to 14CHDO transformation (respectively, 2.9 min^−1^ ↔ 3.73 × 10^−10^ min^−1^) (Fig. 3F and E) and the reaction rates of the 2A to 4A and 2A to HQ reactions (respectively, 4.57 × 10^−3^ min^−1^ and 6.01 × 10^−3^ min^−1^) (Fig. 3C). These two observations combined with the notion that the transformation of 3A to 4A (14CHDO) is very slow (Fig. 3D) establish the preferred reaction mechanism as DMBQ → 2A → HQ → 14CHDO. Details of the determination of the rate-determining steps can be found in ESI section 2.3.†

### Catalytic demethoxylation and hydrogenation of the lignin-derived DO fraction to 14CHDO

**Fig. 4:**
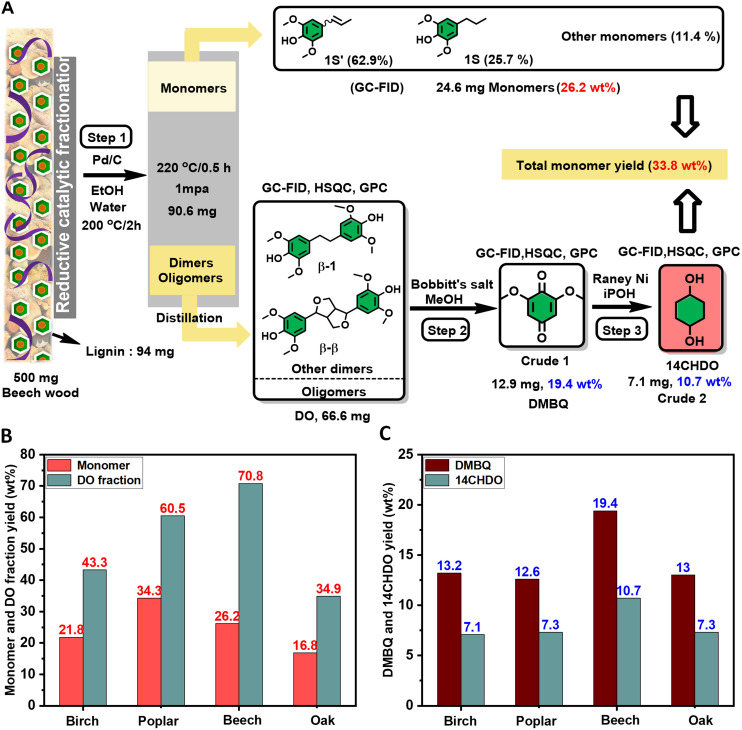
An overview toward the production of 14CHDO from different types of lignocellulose. (A) Step 1: RCF of beech wood produced crude lignin oil (500 mg beech wood, 50 mg Pd/C catalyst, 12 mL ethanol/12 mL water, 200 °C, 2 h) followed by DCM/water partitioning to eliminate the saccharide content; Step 2: Catalytic oxidative deconstruction of the dimeric and oligomeric lignin fraction into **DMBQ** using Bobbitt's salt. (66.6 mg **DO** fraction, 264 mg Bobbitt's salt, 7 ml methanol, 0.4 wt% water relative to methanol, 100 °C, 0.5 h); Step 3: Catalytic demethoxylation and hydrogenation of Crude 1 into **14CHDO** over RANEY® Ni catalyst. (Crude 1, 200 mg RANEY® Ni, 15 mL isopropanol, 30 bar H_2_, 170 °C, 10 h). The red-colored yield values were determined on a lignin basis. The blue-colored yield values labeled refer to the **DO** fraction; (B) monomer and **DO** yield were obtained from different types of wood biomass; (C) DMBQ and 14CHDO yields were obtained from different wood biomass.

Having identified the optimal reaction conditions, their validity was tested towards the conversion of the oligomers in a real lignin oil obtained from beech wood, a hardwood species. To this end, beech wood was converted into a suitable lignin oil by following a published procedure by Samec.^16^ The details of this procedure are explicitly outlined in ESI section 2.5† and are also depicted in Fig. 4A. Importantly, the oil was subjected to DCM/water partitioning, thereby removing the saccharides, followed by fractional distillation of the DCM phase (1 mbar, 220 °C, 0.5 h). The latter operation resulted in two distinct fractions, namely: (1) low molecular-weight lignin-derived monomers, predominantly with 2,6-dimethoxy-4-(propenyl)phenol (1S′) and 4-propylsyringol (1S) signature (total 24.6 mg) and (2) a higher molecular-weight fraction predominantly displaying β-1 and β–β linkages (shown in HSQC – Fig. S14†) and containing mainly lignin dimers and oligomers (total 66.6 mg). The analysis of the latter fraction was also performed by GC-FID/MS (Fig. S9†) and GPC (Fig. S12†), with the latter characterization technique revealing an average oligomer molecular weight (*M*_w_) of 1480 g mol^−1^. Catalytic oxidative cleavage of the dimeric and oligomeric lignin fractions was performed using commercially available Bobbitt's salt, thereby introducing a relevant amount of DMBQ into the lignin oil *i.e.*, 19.4 wt% DMBQ *vis-à-vis* the original dimeric and oligomeric lignin fractions. Eliminating all reduced Bobbitt's salt *via* filtration by means of a short silica gel column (1 vol% MeOH in DCM) gave Crude 1, the characterization of which was performed by GC-FID/MS (Fig. S10†), GPC (*M*_w_ = 321 g mol^−1^, *M*_n_ = 189 g mol^−1^) (Fig. S13†) and HSQC (Fig. S15†). This concisely showed the decomposition of the lignin-derived oligomers into DMBQ. Using the here developed RANEY® Ni catalysed methodology, it was possible to demethoxylate/hydrogenate the DMBQ contained in Crude 1, yielding 14CHDO (10.7 wt%) containing Crude 2. Characterization of Crude 2 was performed by GC-FID/MS (Fig. S11†) and HSQC (Fig. S16†), thereby unequivocally confirming the conversion of DMBQ to 14CHDO.

To illustrate the generality of this methodology, birch, poplar, and oak lignocellulose (500 mg each) were also subjected to the same catalytic protocol treatment (Fig. 4B), giving the respective 14CHDO yields of 7.1 wt%, 7.3 wt% and 7.3 wt% on DO basis as shown in Fig. 4C.

### Catalytic direct amination of 14CHDO to 14CHDA

**Fig. 5:**
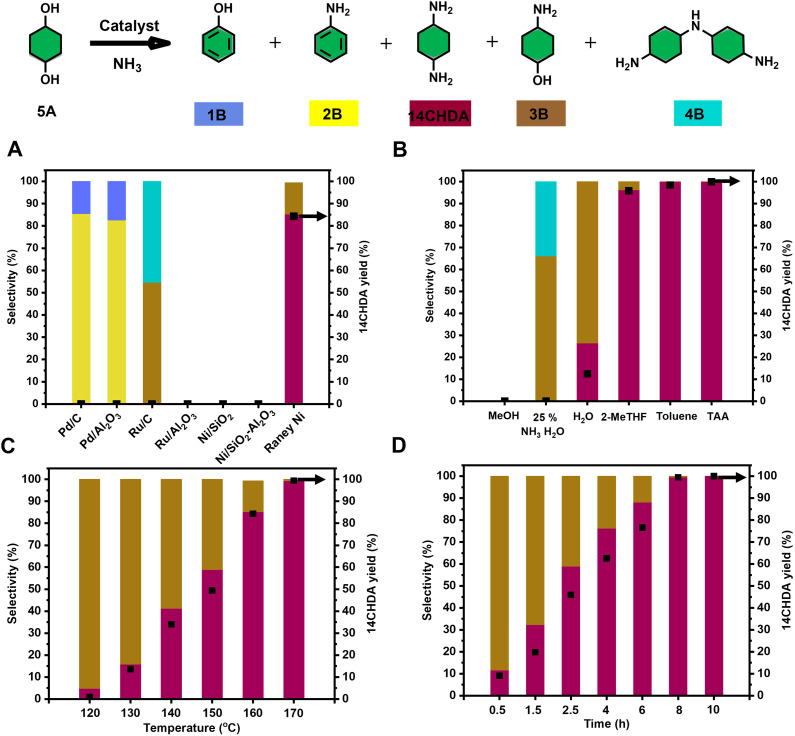
Optimization of the reaction conditions for the catalytic conversion of 14CHDO to 14CHDA (A–D); Unless otherwise specified, the standard reaction conditions are: 14CHDO (0.5 mmol), 100 mg catalyst, 2.5 mL solvent, 7 bar NH_3_, 5 mg dodecane. (A) Screening of a range of commercially available heterogeneous metal catalysts at 160 °C for 3 h; (B) different solvents (150 °C, 8 h); (C) reaction temperature, 3 h; (D) reaction time, 150 °C; (A–D). Detailed numerical values are available in the ESI and more specifically in Tables S10–S13;† the conversion and yield values are determined by GC-FID using calibration curves and the internal standard; the black square in the figures refers to the 14CHDA yield.

Next, the catalytic direct coupling of 14CHDO with ammonia to 14CHDA*via* a hydrogen-borrowing methodology was investigated. Firstly, the influence of a range of commercially available (heterogeneous) metal catalysts was assessed. It was found that Ru and Ni based catalysts display no meaningful reactivity towards this transformation, while Pd catalysts actively engaged in the formation of aniline (2B) (>80% selectivity) over the target product 14CHDA (Fig. 4A, and Table S11†) and phenol (1B). Uniquely, of all tested commercially available heterogeneous metal catalysts, only the use of RANEY® Ni led to the formation of 14CHDA in high yield (up to 85%), the main side product being the mono-aminated product 4-aminocyclohexanol (3B – 15% yield). This unique catalytic ability of RANEY® Ni was attributed to its distinct capability of engaging in all the relevant reaction steps to the hydrogen borrowing sequence: alcohol dehydrogenation and imine reduction. High-loading of catalyst is required because RANEY® Ni occurs as a mixture of metallic nickel and inactive nickel oxide nickel as well as inaccessible Ni in the bulk of the catalyst. Also, commercial wet RANEY® nickel used in the here-presented amination contains a substantial amount of water (∼20 wt%). Surprisingly though, isolation of 14CHDA from such a mixture (mainly 14CHDA and 3B) by either column chromatography or (HCl) salt formation was found to be particularly challenging. Fortunately, dedicated optimization of the reaction temperature (Fig. 4C and Table S13†) and the reaction time (Fig. 4D and Table S14†) revealed conditions (170 °C and 8 h) under which 14CHDA was formed in near quantitative yield. From a solvent perspective, it could be further established that the use of *t*-amyl alcohol and toluene was most beneficial for the 14CHDO to 14CHDA transformation (Fig. 4B and Table S12†). As observed during the 14CHDA formation, the use of MeOH as the solvent gave no noticeable conversion. Using water as the solvent gave a mixture of 14CHDA (25%) and 4B (75%) with a conversion of 64%. The specific use of 25 wt% NH_3_ in H_2_O led to the preferential formation of the mono-aminated product 3B and the 4B dimer in line with the literature.^37,55,56^ Of particular interest is the distinct change in isomeric ratio upon going from 14CHDO to 14CHDA. Indeed, while 14CHDO with a *cis*/*trans* 58 : 42 mixture was subjected to amination, 14CHDA was obtained with a 25 : 75 *cis*/*trans* ratio, which is different from commercially available 14CHDA with a 75 : 25 *cis*/*trans* ratio as also explained in more detail in ESI Note 4.† It is noteworthy to mention that *trans*14CHDA is more stable than *cis*14CHDA. Interestingly, a clear effect on the thermal behavior, such as melting temperature and crystallization temperature, was evidenced with the use of the pure isomer for the preparation of polyamides.^26^ After filtration and solvent removal, 14CHDA was isolated in an 86% yield and characterized by ^1^H NMR and ^13^C NMR (Fig. S19 and S20†) (Fig. 5).

## Conclusion

In conclusion, this work presents a catalytic strategy to valorize the hereto challenging RCF lignin-derived oligomeric fraction, featuring distinct C–C linkages, and convert it to industrially relevant 14CHDO and further on to 14CHDA. To this end, this work takes maximum advantage of a previously developed procedure by the group of Samec for the conversion of the RCF lignin-derived oligomeric fraction into DMBQ—a new platform molecule derivable from lignin.^16^ Additionally, it is concisely shown that commercially available RANEY® Ni can uniquely catalyze both the DMBQ to 14CHDO transformation and the latter's diamination to 14CHDA. This is an important finding as it provides a new sustainable route to a set of compounds, 14CHDO and 14CHDA, which are both highly industrially relevant and cover a multitude of industries and applications. More specifically, this is achieved by a noble-metal-free catalytic procedure comprising of the following two steps: (a) the catalytic demethoxylation and hydrogenation of DMBQ into 14CHDO (86% yield) and (b) the quantitative catalytic direct amination of 14CHDO with ammonia to 14CHDA. Most notably, these catalytic steps can all be conveniently catalyzed by RANEY® Ni—a true workhorse in the current chemical industry. Furthermore, this procedure is shown to be applicable in the conversion of a wider range of oligomeric lignin streams, as generally present in crude (RCF) lignin oils. Overall, the here presented catalytic procedure is capable of transforming high molecular weight lignin oil of beech wood into 10.7 wt% of 14CHDO on a DO basis. It is noteworthy that the here developed methodology does not derive 14CHDO and 14CHDA from petroleum-derived resources and in particular carcinogenic/mutagenic benzene, therefore providing another stride to a more sustainable chemical provision.

## Conflicts of interest

The authors declare no competing interests.

## Supplementary Material

GC-025-D2GC03777G-s001
