# Supplementary material for: High yield production of 1,4-cyclohexanediol and 1,4-cyclohexanediamine from high molecular-weight lignin oil
Source: Green Chem. 2022 Nov 29;25(1):211–20. doi: 10.1039/d2gc03777g (PMC9808896; doi:10.1039/d2gc03777g)
Supplement: GC-025-D2GC03777G-s001 [file GC-025-D2GC03777G-s001.pdf]

Electronic Supplementary Material (ESI) for Green Chemistry.
This journal is © The Royal Society of Chemistry 2022

Supplementary information

High yield production of 1,4-Cyclohexanediol and 1,4-Cyclohexanediamine from High Molecular-weight Lignin oil

Xianyuan Wu[a], Mario De bruyn[c], Julia Michaela Hulan[c], Henrique Brasil[c], Zhuohua Sun[b] and Katalin Barta*[a,c]

[a]Stratingh Institute for Chemistry, University of Groningen, Groningen, The Netherlands.

[b]Beijing Key Laboratory of Lignocellulosic Chemistry, Beijing Forestry University, No. 35 Tsinghua East Road Haidian District, Beijing, 100083, P. R. China.

[c]Department of Chemistry, Organic and Bioorganic Chemistry, University of Graz, Heinrichstrasse 28/II, 8010 Graz, Austria

*Correspondence to: katalin.barta@uni-graz.at

Table of Contents

1. General information 1

1.1 Preparation of model compounds 2

1.2 General experimental procedure 2

2. Catalytic demethoxylation and hydrogenation of DMBQ to 14CHDO over Raney Ni catalyst. 3

2.1 GC-FID traces of catalytic demethoxylation and hydrogenation of DMBQ to 14CHDO over Raney Ni catalyst 3

2.2 Establishing the optimal reaction conditions for the catalytic demethoxylation and hydrogenation of DMBQ to 14CHDO 4

2.3 Reaction kinetics for catalytic demethoxylation/hydrogenation of DMBQ to 14CHDO over Raney Ni catalyst 5

2.4 Determination of cis and trans isomers and ratio in 14CHDO obtained from catalytic demethoxylation and hydrogenation of DMBQ 12

2.5 A sustainable pathway for the production of 14CHDO from native lignocellulose 14

2.5.1 GC-FID/MS traces of monomer and DO, Crude 1 and Crude 2 fraction 16

2.5.2 GPC traces of monomer and DO and Crude 1 fraction 17

2.5.3 2D HSQC spectra of DO, Crude 1 and Crude 2 fraction 18

3. Catalytic direct amination of 14CHDO with ammonia to 14CHDA over Raney Ni catalyst 20

3.1 Detailed analysis of crude 14CHDA obtained from catalytic amination of 14CHDO 20

3.2 Establishing the optimal reaction conditions for catalytic direct amination of 14CHDO to 14CHDA 22

4. Reference 23

Supplementary Note 1: Proposed industrial pathways for 14CHDO and 14CHDA production ... 24

Supplementary Note 2: Considerations for the high catalytic performance of Raney Nickel catalyst for catalytic demethoxlation and hydrogenation of DMBQ 24

1. General information

Column chromatography was performed using Merck silica gel type 9385 230–400 mesh and typically dichloromethane and methanol or EtOAc and pentane as eluent.

Thin layer chromatography (TLC): Merck silica gel 60, 0.25 mm. The components were visualized by UV or $KMnO_4$ staining.

Gas Chromatography (GC) was used for product identification as well as determination of conversion and selectivity values. Product identification was performed by GC-MS (5975C MSD) equipped with an HP-5 MS column, and helium as carrier gas. The temperature program started at 50 °C for 5 min, heated by 10 $°C·min^{-1}$ to 325 °C and held for 5 min. Conversion and products selectivity were determined by GC-FID (Agilent 8890 GC) equipped with an HP-5MS column using nitrogen as carrier gas.

For the analysis of high molecular-weight fraction of lignin oil[1]: A 2mL GC vial was charged with 10 mg of **DO**, 1 mL anhydrous DCM, 50 μL of anhydrous pyridine and 100 μL of BSTFA. The vial was placed in an oven, and was kept at 60 °C for 1 hour. After that, the mixture was then subjected to GC-FID/MS analysis.

Nuclear Magnetic Resonance (NMR) spectroscopy:

^1H, and ^{13}C NMR spectra were recorded on a Bruker Avance III 300 MHz (300 and 75 MHz, respectively) and 2D NMR spectra were recorded on a Bruker Avance III 700 MHz with Cryoplatform and a 5mm Triple-Resonance cryoprobe (700 and 175 MHz, respectively). ^1H,^{13}C NMR and 2D NMR spectra were recorded at RT. Chemical shift values are reported in ppm with the solvent resonance as the internal standard ($CDCl_3$: 7.26 for ^1H, 77.0 for ^{13}C; CD_3OD: 3.31 for ^1H, 49.0 for ^{13}C; DMSO-d_6: 2.50 for ^1H, 39.5 for ^{13}C). Data are reported as follows: chemical shifts, multiplicity (s = singlet, d = doublet, t = triplet, q = quartet, br. = broad, m = multiplet), coupling constants (Hz), and integration.

Gel Permeation Chromatography (GPC) GPC was performed at the University of Graz on a SHIMADZU NEXERA equipped 2×SDV analytical Linear M (8×300 mm, 5μm) plus 1×precolumn SVD (8×50mm, 5μm). The columns were operated at 40 °C with a flow-rate of 1 $mL·min^{-1}$ of THF. Detection was accomplished at 40 °C using an SPD-M40 photoarray detector in series. The molecular weight estimations were performed using polystyrene standards of known molecular weight distribution.

Abbreviations

DMBQ: 2,6-dimethoxybenzoquinone

1A: 2,6-dimethoxybenzene-1,4-diol

2A: 2-methoxybenzene-1,4-diol

3A: 2,6-dimethoxycyclohexane-1,4-diol

4A: 2-methoxycyclohexane-1,4-diol

14CHDO: 1,4-cyclohexanediol

5A: cyclohexanol

6A: cyclohexane

1B: phenol **2B**: aniline **14CHDA**: 1,4-cyclohexanediamine **3B**: 4-aminocyclohexanol

4B: (4-aminocyclohexyl)cyclohexane-1,4-diamine

β-1: 4,4'-(ethane-1,2-diyl)bis(2,6-dimethoxyphenol) **β-β**: syringaresinol

1.1 Preparation of model compounds

Preparation of 2,6-dimethoxycyclohexane-1,4-diol (3A): In a typical procedure, a 100 mL high-pressure Parr autoclave was charged with 100 mg Pd/C catalyst, 500 mg 2,6-dimethoxybenzene-1,4-diol, 20 mL 2-Me THF, and equipped with mechanical stirring. The reactor was sealed and purged 3 times with H_2 and then pressurized with H_2 (40 bar). The reactor was heated to 140 °C for 6 h under stirring at 400 rpm. After completion of the reaction, the reactor was cooled to RT. Then the product was purified by silica gel column chromatography (gradient elution: methanol: dichloromethane: 0.5: 99.5- 2: 98). Finally, 390 mg transparent viscous liquid (**3A**) containing isomers in a purity of 96 %, characterized by GC was obtained in a yield of 75.6 %.

Preparation of 2-methoxycyclohexane-1,4-diol (4A): In a typical procedure, a 100 mL high-pressure Parr autoclave was charged with 100 mg Pd/C catalyst, 500 mg 2-methoxybenzene-1,4-diol, 20 mL 2-Me THF, and equipped with mechanical stirring. The reactor was sealed and purged 3 times with H_2 and then pressurized with H_2 (40 bar). The reactor was heated to 140 °C for 6 h under stirring at 400 rpm. After completion of the reaction, the reactor was cooled to RT. Then the crude product (496 mg) was collected in a yield of 95.2 % after removing the 2-Me THF under reduced pressure. Then 0.1 mL solution was collected through a syringe and injected into GC-FID after filtration through a PTFE filter (0.45 µm), which shows more than 95 % purity to 2-methoxycyclohexane-1,4-diol (**4A**).

1.2 General experimental procedure

Catalytic demethoxylation and hydrogenation of 2,6-dimethoxybenzoquinone (DMBQ) into 14CHDO: The catalytic demethoxylation/hydrogenation of **DMBQ** was carried out in a 100 mL high-pressure Parr autoclave equipped with an overhead stirrer. Typically, the autoclave was charged with 200 mg Raney Ni catalyst, 1 mmol 2,6-dimethoxybenzoquinone, 20 mL isopropanol and 10 mg dodecane as internal standard. The reactor was sealed and purged 3 times with H_2 and then pressurized with H_2 (30 bar). The reactor was then heated to the desired temperature and stirred at 400 rpm for 4 h. After the reaction was completed, the reactor was cooled down to RT. Then 0.1 mL solution was collected through a syringe and injected to GC-MS or GC-FID after filtration through a PTFE filter (0.45 µm).

The catalytic direct amination of 14CHDO into 14CHDAover Raney Ni catalyst with ammonia gas: The catalytic direct amination of **14CHDO** into **14CHDA** was performed in 10 mL high-pressure autoclave equipped with magnetic stirring bar. Typically, a 4 mL vial was charged with 100 mg Raney Ni catalyst, 0.5 mmol **14CHDO**, 2.5 mL *t*-amyl alcohol, 5 mg dodecane as an internal standard. Then the vial was sealed inside autoclave and pressurized with 7 bar NH_3. The reactor was heated to 150 °C and stirred at 400 rpm for 18 h. After completion of the reaction, the reactor was cooled down to RT. Then, 0.1 mL solution was collected through a syringe and injected to GC-MS or GC-FID after filtration through a PTFE filter (0.45 µm). The crude mixture was then subjected to characterizations d by ^1H and ^{13}C-NMR.

2. Catalytic demethoxylation and hydrogenation of DMBQ to 14CHDO over Raney Ni catalyst

2.1 GC-FID traces of catalytic demethoxylation and hydrogenation of DMBQ to 14CHDO over Raney Ni catalyst

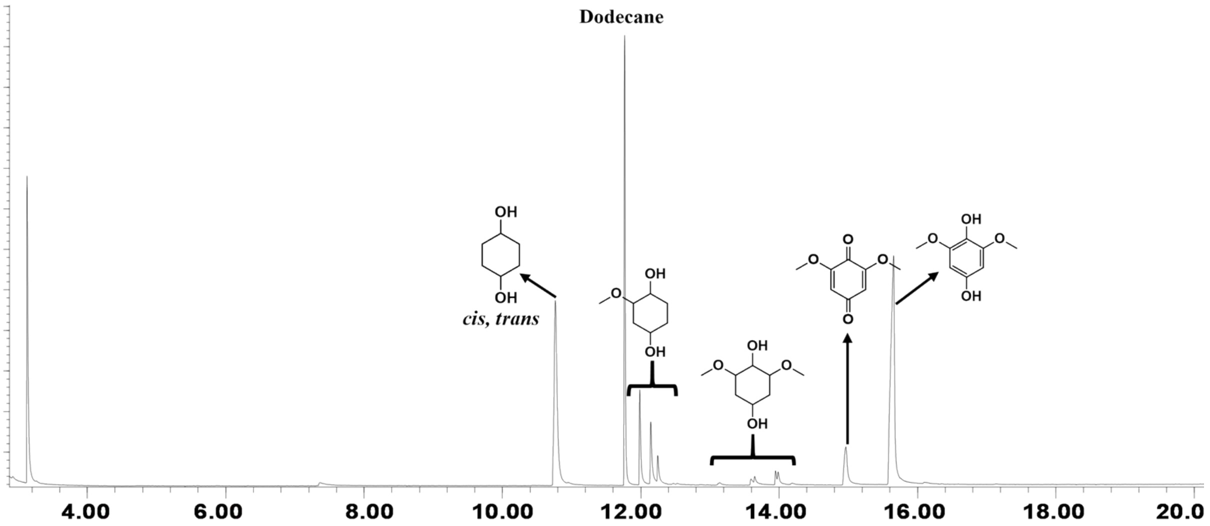

Figure S1. Catalytic demethoxylation and hydrogenation of **DMBQ** to **14CHDO** over Raney Ni catalyst. (Reaction conditions: 1 mmol **DMBQ**, 200 mg Raney nickel, 15 mL isopropanol, 10 mg dodecane, 170 °C, 10 min)

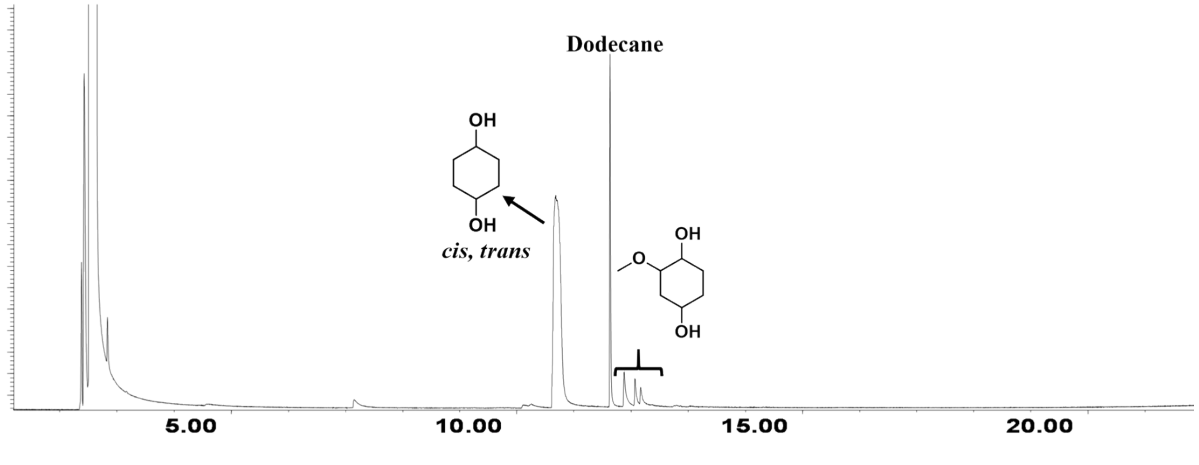

Figure S2. Catalytic demethoxylation and hydrogenation of **DMBQ** to **14CHDO** over Raney Ni catalyst. (Reaction conditions: 1 mmol **DMBQ,** 200 mg Raney nickel, 15 mL isopropanol, 10 mg dodecane, 170 °C, 10 h)

2.2 Establishing the optimal reaction conditions for the catalytic demethoxylation and hydrogenation of DMBQ to 14CHDO

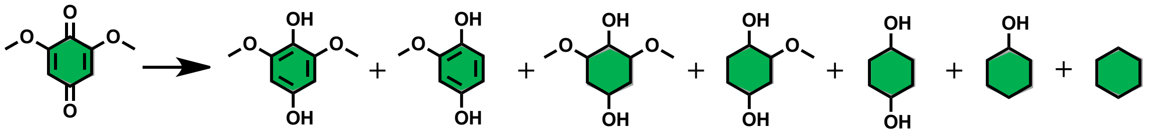

DMBQ **1A** **2A** **3A** **4A** **14CHDO** **5A** **6A**

Table S1. Survey of catalysts for catalytic demethoxylation and hydrogenation of **DMBQ**[a]

Catalyst	Conv. (%)	Sel. (%)						Yield (%)
		**3A**	**4A**	**14CHDO**	**5A**	**6A**	Others	
Raney nickel	100	2.9	30.2	65.9	1.0	0	0	65.9
Pd/C	100	86.4	9.2	4.4	0	0	0	4.4
Pd/Al_2O_3	100	71.6	16.2	12.2	0	0	0	12.2
Ru/C	100	12.6	42.2	31.7	6.0	0	7.5	31.7
Ru/Al_2O_3	100	9.2	20.5	55.3	15.0	0	0	55.3
Ni/SiO_2	100	10.2	36.8	50.4	2.6	0	0	50.4
Ni/SiO_2-Al_2O_3	100	14.6	44.2	39.4	1.5	0.3	0	39.4

[a]. Reaction conditions: **DMBQ** (1 mmol, 0.168 g), 200 mg catalyst, 170 °C, 4 h, 15 mL isopropanol, 10 mg dodecane; Conversion, selectivity and yield values determined by GC-FID using calibration curves and internal standard;

Table S2. Solvent influence in the catalytic demethoxylation and hydrogenation of **DMBQ**[a]

Solvent	Conv. (%)	Sel. (%)								Yield (%)
		**1A**	**2A**	**3A**	**4A**	**14CHDO**	**5A**	**6A**	others	
Water	100	13.0	0	9.8	21.2	52.8	3.1	0	0	52.8
Methanol	88.0	100	0	0	0	0	0	0	0	0
Ethanol	89.1	40.4	0	8.4	16.7	12.8	0	0	21.7	11.4
Propanol	91.0	9.5	0	21.7	39.4	24.9	1.9	2.6	0	22.7
Isopropanol	100	0	0	2.9	30.2	65.9	1.0	0	0	65.9
2-Me-THF	96.0	0	7.3	0	15.5	63.4	4.9	3.6	5.3	60.9
t-amyl alcohol	100	0	0	0	25.2	62.0	2.4	1.4	9.0	62.0

[a]. Reaction conditions: **DMBQ** (1 mmol, 0.168 g), 200 mg Raney Ni, 170 °C, 4 h, 15 mL solvent, 10 mg dodecane; Conversion, selectivity and yield values determined by GC-FID using calibration curves and internal standard;

Table S3. Influence of reaction temperature for catalytic demethoxylation and hydrogenation of **DMBQ**[a]

Temperature (°C)	Conv. (%)	Sel. (%)						Yield (%)
		**1A**	**3A**	**4A**	**14CHDO**	**5A**	**6A**	
140	0.95	8.9	12.2	44.7	34.2	0	0	32.5
150	0.98	8.1	11.0	43.0	37.9	0	0	37.1
160	100	0	8.1	43.5	48.4	0	0	48.4
170	100	0	3.4	37.6	58.2	0.8	0	58.2
180	100	0	2.7	30.7	64.9	1.7	0	64.9
190	100	0	0	8.8	85.2	4.8	1.2	85.2

[a]. Reaction conditions: **DMBQ** (1 mmol, 0.168 g), 200 mg Raney Ni, 140-190 °C, 2 h, 15 mL isopropanol, 10 mg dodecane; Conversion, selectivity and yield values determined by GC-FID using

calibration curves and internal standard;

Table S4. Influence of reaction time for catalytic demethoxylation and hydrogenation of **DMBQ**[a]

Time (h)	Conv. (%)	Sel. (%)						Yield (%)
		**1A**	**3A**	**4A**	**14CHDO**	**5A**	**6A**	
0.17	93.5	51.0	2.5	18.8	27.7	0	0	25.9
0.33	94.5	25.9	4.0	29.6	40.5	0	0	38.3
1	100	0	2.9	37.7	59.4	0	0	59.4
3	100	0	1.4	34.1	63.6	0.9	0	63.6
6	100	0	1.3	25.2	72.1	1.4	0	72.1
10	100	0	0	15.0	83.3	1.7	0	83.3
12	100	0	0	8.7	86.3	3.7	1.3	86.3
14	100	0	0	4.2	84.0	7.8	4.0	83.9

[a]. Reaction conditions: **DMBQ** (1 mmol, 0.168 g), 200 mg Raney Ni, 170 °C, 0-14 h, 15 mL isopropanol, 10 mg dodecane; Conversion, selectivity and yield values determined by GC-FID using calibration curves and internal standard;

2.3 Reaction kinetics for catalytic demethoxylation/hydrogenation of DMBQ to 14CHDO over Raney Ni catalyst

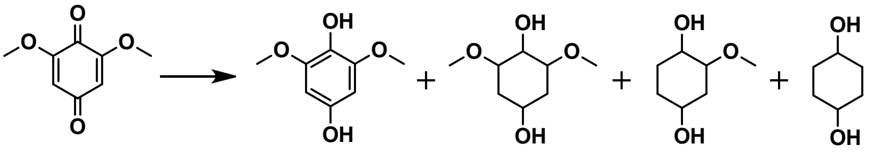

DMBQ **1A** **3A** **4A** **14CHDO**

Table S5. Reaction kinetic data for catalytic demethoxylation and hydrogenation of **DMBQ**[a]

Time (min)	Conversion (%)	**DMBQ** Concentration (mmol/ml)	Concentration (mmol/ml)			
			**1A**	**3A**	**4A**	**14CHDO**
0	0	0.075	0	0	0	0
10	50.6	0.03705	0.03165	0.000247	0.003016	0.003036
20	64.1	0.02691	0.03995	0.000542	0.003742	0.003812
40	73.1	0.020175	0.041969	0.001609	0.006032	0.005215
60	80.2	0.01485	0.042737	0.002352	0.008282	0.006779
120	87.4	0.00945	0.033816	0.005351	0.015002	0.01138
240	91.5	0.006375	0.019617	0.007794	0.023267	0.017947
360	94.9	0.003825	0.009066	0.009215	0.029184	0.023709
480	96.1	0.002925	0.005733	0.009863	0.030755	0.025724

[a]. Reaction conditions: 1.5 mmol **DMBQ**, 30 mg catalyst, 170 °C, 30 bar H_2, 20 mL isopropanol, 10 mg dodecane; Conversion and concentration determined by GC-FID using calibration curves and internal standard.

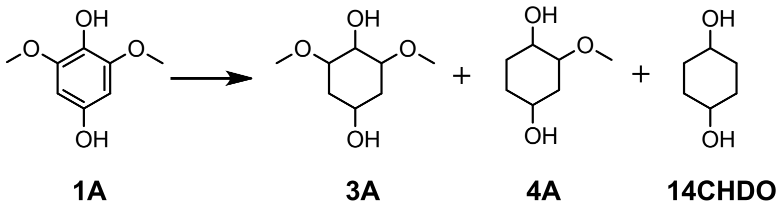

Table S6. Reaction kinetic data for catalytic demethoxylation and hydrogenation of **1A**[a]

Time (min)	Conversion (%)	**1A** Concentration (mmol/ml)	Concentration (mmol/ml)		
			**3A**	**4A**	**14CHDO**
0	0	0.075	0	0	0
10	3.5	0.0723	0.0001119	0.001122	0.001391
20	6.1	0.0704	0.000196	0.002014	0.002364
40	12.1	0.06592	0.000932	0.003852	0.00429
60	19.1	0.060675	0.001843	0.006158	0.006323
120	37.4	0.04695	0.00447	0.012438	0.01114
180	50.8	0.0369	0.006412	0.016779	0.014908
240	63.1	0.02745	0.008212	0.021084	0.018254
360	77.8	0.01665	0.00972	0.026557	0.022072
480	96.1	0.002925	0.011288	0.032546	0.0282399
600	97.5	0.001875	0.010927	0.032743	0.0294547

[a]. Reaction conditions: 1.5 mmol **1A**, 30 mg catalyst, 170 °C, 30 bar H_2, 20 mL isopropanol, 10 mg dodecane; Conversion and concentration determined by GC-FID using calibration curves and internal standard.

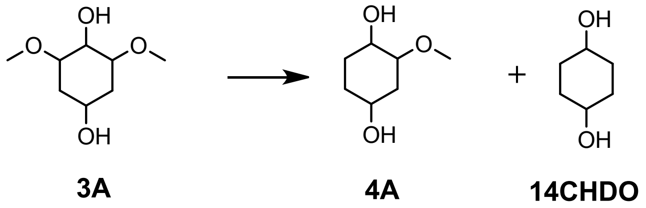

Table S7. Reaction kinetic data for catalytic demethoxylation and hydrogenation of **3A**[a]

Time (min)	Conversion (%)	**3A** Concentration (mmol/ml)	Concentration (mmol/ml)	
			**4A**	**14CHDO**
0	0	0.05	0	0
10	1.8	0.0491	0.00044	0.0004603
30	2.2	0.0489	0.000556	0.0005435
60	2.5	0.04875	0.000645	0.0006048
90	2.7	0.04865	0.000702	0.000648
120	2.9	0.04855	0.000762	0.0006877
180	3.3	0.04835	0.000888	0.0007619
240	3.7	0.04815	0.001001	0.000849
300	3.9	0.04805	0.001054	0.0008959
360	4.2	0.0479	0.001154	0.000946
480	4.6	0.0477	0.001286	0.001014

[a]. Reaction conditions: 1 mmol **3A**, 15 mg catalyst, 170 °C, 30 bar H_2, 20 mL isopropanol, 10 mg dodecane; Conversion and concentration determined by GC-FID using calibration curves and internal standard.

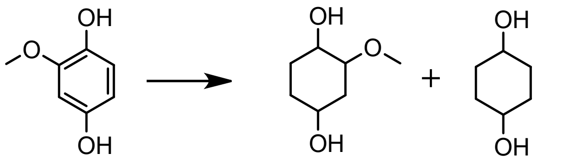

2A **4A** **14CHDO**

Table S8. Reaction kinetic data for catalytic demethoxylation and hydrogenation of **2A**[a]

Time（min）	Conversion (%)	**2A** Concentration (mmol/ml)	Concentration (mmol/ml)	
			**4A**	**14CHDO**
0	0	0.075	0	0
10	8.0	0.069	0.003576	0.002424
20	13.8	0.06465	0.005574	0.004776
40	31.0	0.05175	0.01264	0.01061
60	45.4	0.04095	0.01861	0.01544
90	59.2	0.0306	0.02486	0.020114
120	77.3	0.017025	0.031472	0.026503
180	89.6	0.0078	0.03679	0.03041
240	94.5	0.004125	0.039543	0.031332
360	96.1	0.002925	0.040873	0.031202
480	96.7	0.002475	0.041634	0.030891
600	98.8	0.0009	0.045716	0.028384

[a]. Reaction conditions: 1.5 mmol **2A**, 30 mg catalyst, 170 °C, 30 bar H_2, 20 mL isopropanol, 10 mg dodecane; Conversion and concentration determined by GC-FID using calibration curves and internal standard.

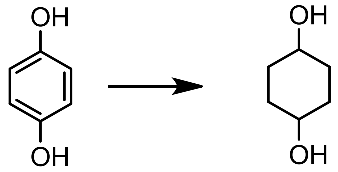

HQ **14CHDO**

Table S9. Reaction kinetic data for catalytic hydrogenation of **HQ**[a]

Time（min）	Conversion (%)	**HQ** Concentration (mmol/ml)	Concentration (mmol/ml)
			**14CHDO**
0	0	0.075	0
10	42.0	0.069	0.01674
20	58.0	0.06465	0.02463
30	72.0	0.05175	0.040366
40	84.0	0.04095	0.05303
50	91.0	0.0306	0.0665457
60	96.0	0.017025	0.07152
70	99.0	0.0078	0.0830

[a]. Reaction conditions: 1.5 mmol **HQ**, 30 mg catalyst, 170 °C, 30 bar H_2, 20 mL isopropanol, 10 mg dodecane; Conversion and concentration determined by GC-FID using calibration curves and internal standard.

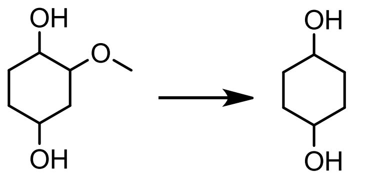

4A **14CHDO**

Table S10. Reaction kinetic data for catalytic demethoxylation and hydrogenation of **4A**[a]

Time (min)	Conversion (%)	**4A** Concentration (mmol/ml)	Concentration (mmol/ml) **14CHDO**
0	0	0.075	0
10	1.9	0.073575	0.001425
40	4.5	0.071625	0.003375
90	5.1	0.071175	0.003825
180	5.4	0.07095	0.00405
240	5.9	0.070575	0.004425
360	6.3	0.070275	0.004725
480	6.9	0.069825	0.005175
600	7.5	0.069375	0.005625

[a]. Reaction conditions: 1.5 mmol **4A**, 30 mg catalyst, 170 °C, 30 bar H_2, 20 mL isopropanol, 10 mg dodecane; Conversion and concentration determined by GC-FID using calibration curves and internal standard.

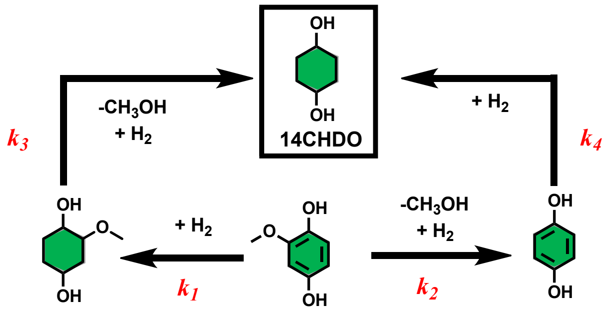

Scheme S1. Proposed reaction network and calculated apparent rate constants

- **Consumption/production equations**

A pseudo-homogeneous kinetics model was used to describe the conversion of 2A into 5A. To simplify the model, the following assumptions were made:

- The concentration of H_2 in liquid phase is constant and the adsorption of H_2 is the most relevant on the catalyst surface.
- The compound 1H is rapidly consumed after its production and was treated as an intermediate, such as:

$$\frac{d[HQ]}{dt} = k_2[2A] - k_4[HQ] = 0$$

- The product 1H was not detected in significant amounts. Thus, $k_4 >> k_2$.

Considering the assumptions detailed above, the model could be simplified to a power-law model, where k_n are the apparent kinetic constants. The resulting system of ordinary differential equations is

the following:

$$\frac{d[2A]}{dt} =- (k_1 + k_2)[2A]$$

$$\frac{d[4A]}{dt} = k_1[2A] - k_3[4A]$$

$$\frac{d[HQ]}{dt} = k_2[2A] - k_4[HQ]$$

$$\frac{d[14CHDO]}{dt} = k_3[4A] + k_4[HQ]$$

- **Matlab input**

The ODE system was input into Matlab and an analytical solution could be found:

Input:

```
syms A(t) B(t) C(t) D(t) k1 k2 k3 k4 A0 B0 C0 D0
eqn1 = diff(A,t) == -k1*A - k2*A;
eqn2 = diff(B,t) == k1*A - k3*B;
eqn3 = diff(C,t) == k2*A - k4*C;
eqn4 = diff(D,t) == k3*B + k4*C;
cond1 = A(0) == A0;
cond2 = B(0) == B0;
cond3 = C(0) == C0;
cond4 = D(0) == D0;
odes = [eqn1;eqn2;eqn3;eqn4];
[ASol(t),BSol(t),CSol(t),DSol(t)] = dsolve(odes,cond1,cond2,cond3,cond4)
```

Solution:

```
ASol(t) = (exp(-t*(k1 + k2))*(A0*k1*k3 + A0*k2*k4 - A0*k3*k4))/(k1*k3 + k2*k4 - k3*k4)

BSol(t) = (exp(-k3*t)*(A0*k1 + B0*k1 + B0*k2 - B0*k3))/(k1 + k2 - k3) - (exp(-t*(k1 + k2))*(k1*k2 - k1*k4 + k1^2)*(A0*k1*k3 + A0*k2*k4 - A0*k3*k4))/((k1*k3 + k2*k4 - k3*k4)*(k1 + k2 - k3)*(k1 + k2 - k4))

CSol(t) = (exp(-k4*t)*(A0*k2 + C0*k1 + C0*k2 - C0*k4))/(k1 + k2 - k4) - (k2*exp(-t*(k1 + k2))*(A0*k1*k3 + A0*k2*k4 - A0*k3*k4))/((k1*k3 + k2*k4 - k3*k4)*(k1 + k2 - k4))

DSol(t) = A0 + B0 + C0 + D0 - (exp(-k3*t)*(A0*k1 + B0*k1 + B0*k2 - B0*k3))/(k1 + k2 - k3) - (exp(-k4*t)*(A0*k2 + C0*k1 + C0*k2 - C0*k4))/(k1 + k2 - k4) + (exp(-t*(k1 + k2))*(A0*k1*k3 + A0*k2*k4 - A0*k3*k4))/((k1 + k2 - k3)*(k1 + k2 - k4))
```

- **Equations and model prediction**

The analytical solution was written in terms of the apparent kinetic constants and concentration of the chemical species.

Compound	Expression
[2A]	$\frac{\left(exp\left(-\left(k_1+k_2\right)t\right)\times\left(k_1k_3[2A]_0+k_2k_4[2A]_0-k_3k_4[2A]_0\right)\right)}{k_1k_3+k_2k_4-k_3k_4}$
[4A]	$\frac{\left(exp\left(-k_3t\right)\times\left(k_1[2A]_0+k_1[4A]_0+k_2[4A]_0-k_3[4A]_0\right)\right)}{k_1+k_2-k_3} - \frac{\left(exp\left(-\left(k_1+k_2\right)t\right)\times\left(k_1k_2-k_1k_4+k_1{}^2\right)\times\left(k_1k_3[2A]_0+k_2k_4[2A]_0-k_3k_4[2.\right.\right.}{\left(\left(k_1k_3+k_2k_4-k_3k_4\right)\times\left(k_1+k_2-k_3\right)\times\left(k_1+k_2-k_4\right)\right)}$
[HQ]	$\frac{\left(exp\left(-k_4t\right)\times\left(k_2[2A]_0+k_1[HQ]_0+k_2[HQ]_0-k_4[HQ]_0\right)\right)}{k_1+k_2-k_4} - \frac{\left(k_2exp\left(-\left(k_1+k_2\right)t\right)\times\left(k_1k_3[2A]_0+k_2k_4[2A]_0-k_3k_4[2A]_0\right)\right)}{\left(\left(k_1k_3+k_2k_4-k_3k_4\right)\times\left(k_1+k_2-k_4\right)\right)}$
[14CHDO]	$[2A]_0+[4A]_0+[1H]_0+[14CHDO]_0-\frac{\left(exp\left(-k_3t\right)\times\left(k_1[2A]_0+k_1[4A]_0+k_2[4A]_0-k_3[4A]_0\right.\right.}{k_1+k_2-k_3} - \frac{\left(exp\left(-k_4t\right)\times\left(k_2[2A]_0+k_1[HQ]_0+k_2[HQ]_0-k_4[HQ]_0\right)\right)}{k_1+k_2-k_4} + \frac{\left(exp\left(-\left(k_1+k_2\right)t\right)\times\left(k_1k_3[2A]_0+k_2k_4[2A]_0-k_3k_4[2A]_0\right)\right)}{\left(k_1+k_2-k_3\right)\times\left(k_1+k_2-k_4\right)}$

The equations described in the table above were used to estimate the concentrations of the chemical species over time. The values of k_1, k_2, k_3 and k_4 were adjusted to minimize the standard deviation between the experimental and predicted points, calculated by the following equation:

$$\sigma=\sqrt{\frac{\sum\left(y_{measured}-y_{predicted}\right)^2}{n-4}}$$

where $y_{measured}$ and $y_{predicted}$ are the measured and predicted concentration for each species, and n is the number of measurements.

Adjustable parameters were determined with σ = 0.004. The graph below shows that the experimental data fit satisfactorily to the first-order pseudo-homogeneous model (dashed line).

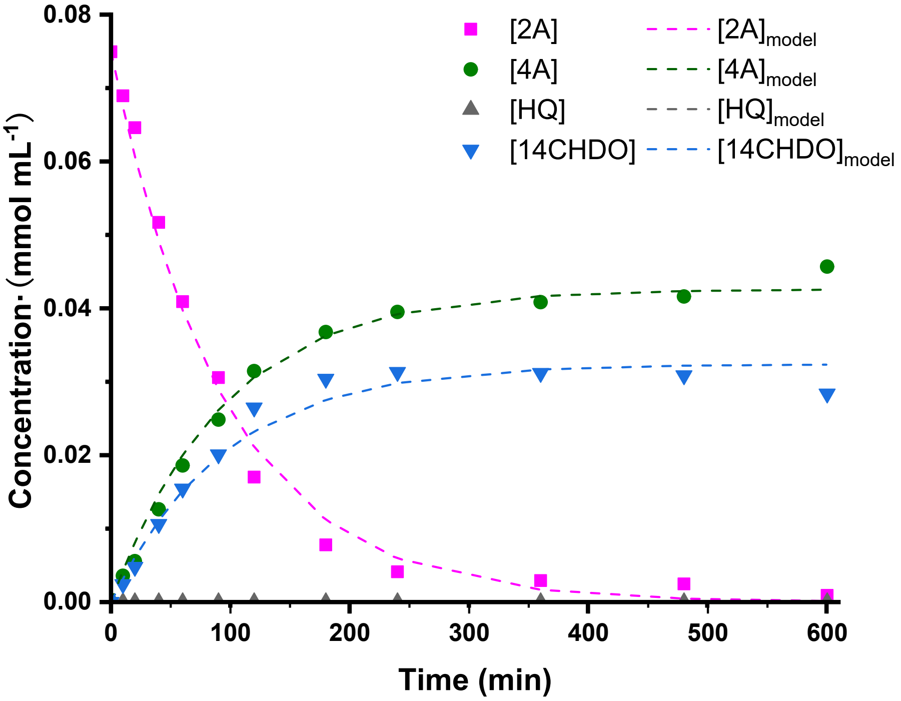

Figure S3. Data fitting spectrum for the demethoxylation and hydrogenation of **2A** over Raney Nicatalyst. Reaction conditions: 1 mmol **2A**, 30 mg Raney nickel, 20 mL isopropanol, 170 °C, 10 mg dodecane, 30 bar H_2.

Finally, the apparent kinetic constants were determined to be the following at 170 °C:

k_1 = 6.01×10^{-3} min^{-1}

k_2 = 4.57×10^{-3} min^{-1}

k_3 = 3.73×10^{-10} min^{-1}

k_4 = 2.90 min^{-1}

2.4 Determination of cis and trans isomers and ratio in 14CHDO obtained from catalytic demethoxylation and hydrogenation of DMBQ

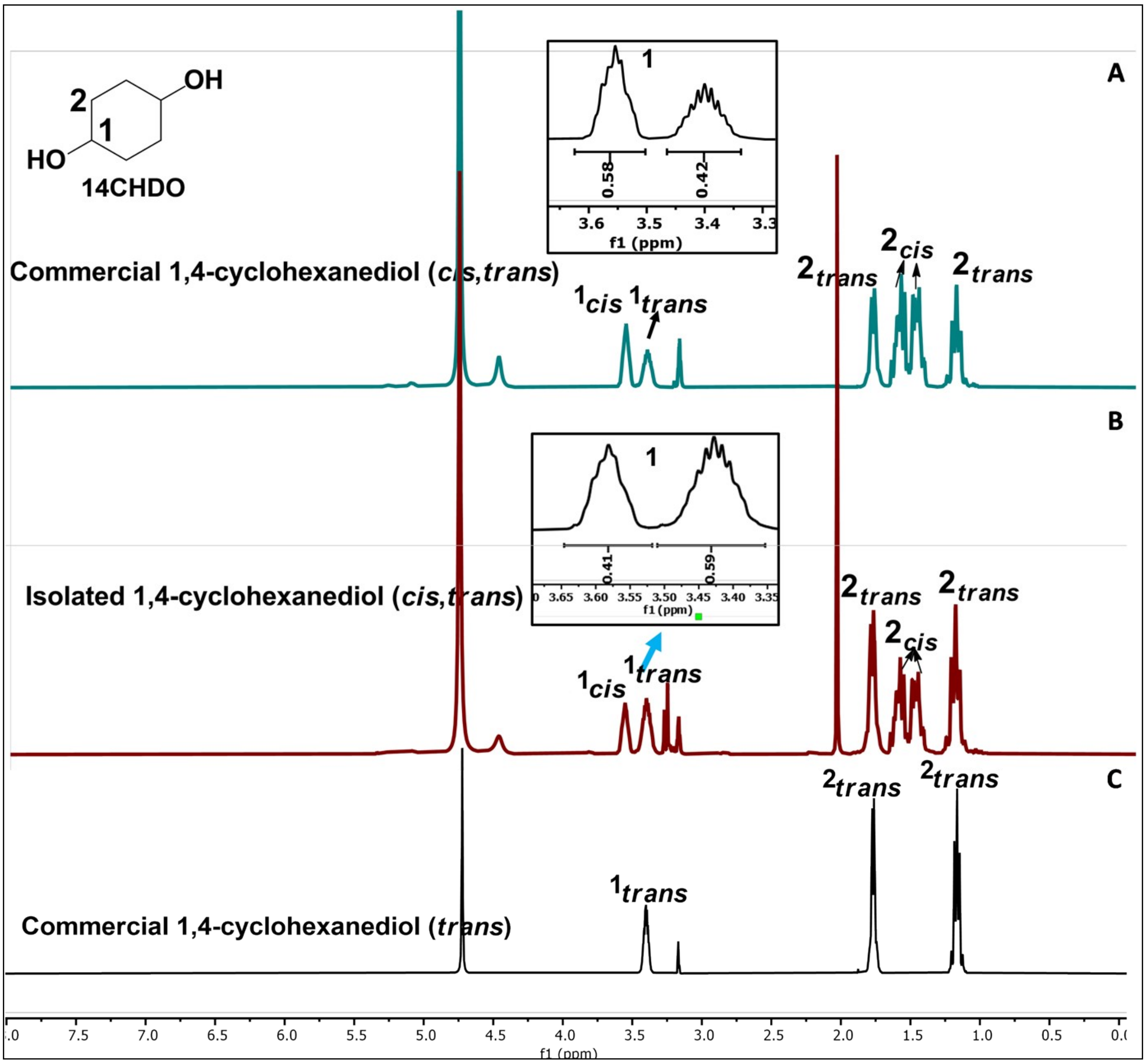

Figure S4. ^{1}H NMR of A) commercial **14CHDO** (cis and trans); B) isolated **14CHDO** (cis and trans) obtained from catalytic demethoxylation and hydrogenation of **DMBQ**; C) commercial **14CHDO** (trans)

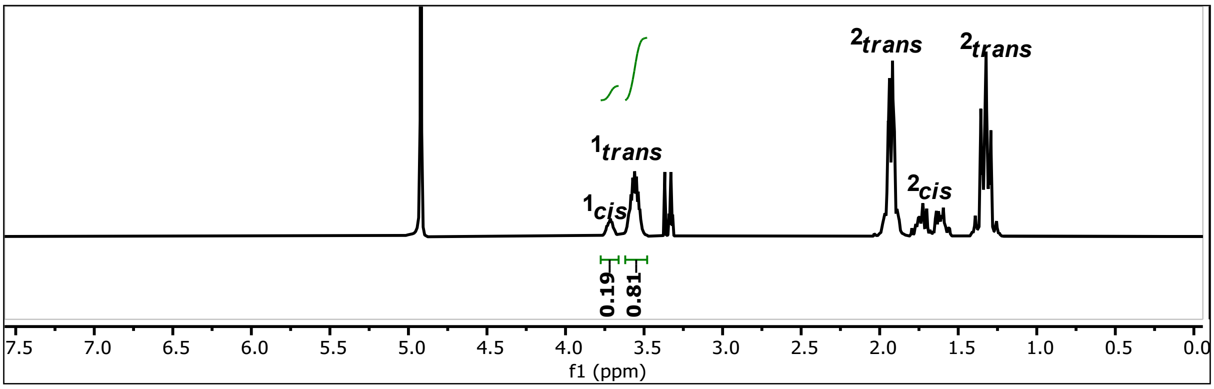

Figure S5. ^{1}H NMR of 8 mg of commercial **14CHDO** (cis and trans) and 25 mg pure **14CHDO** (trans)

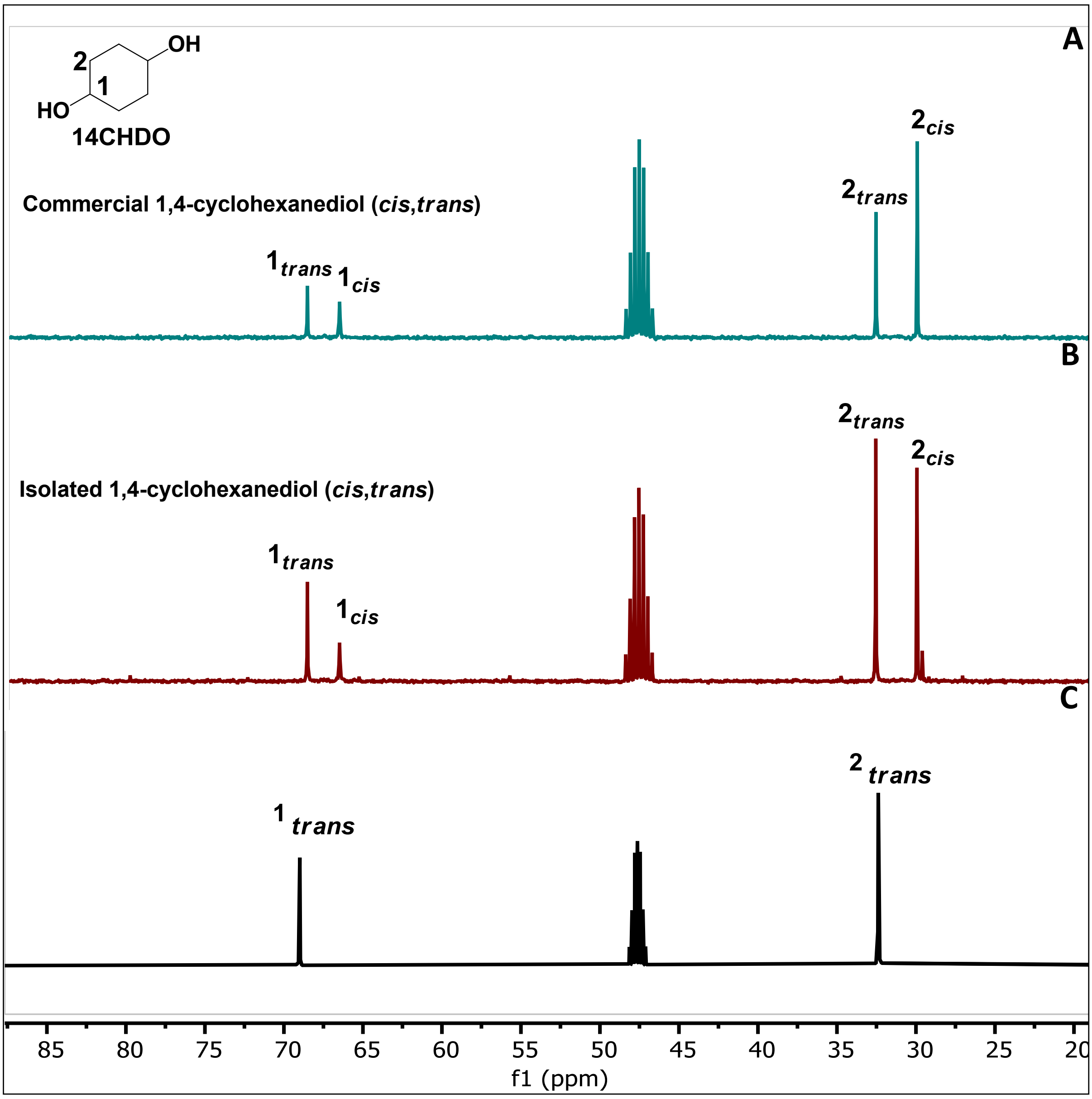

Figure S6. ^{13}C NMR of A) commercial **14CHDO** (cis and trans); B) isolated **14CHDO** (cis and trans) obtained from catalytic demethoxylation and hydrogenation of **DMBQ**; C) commercial **14CHDO** (trans)

2.5 A sustainable pathway for the production of 14CHDO from native lignocellulose

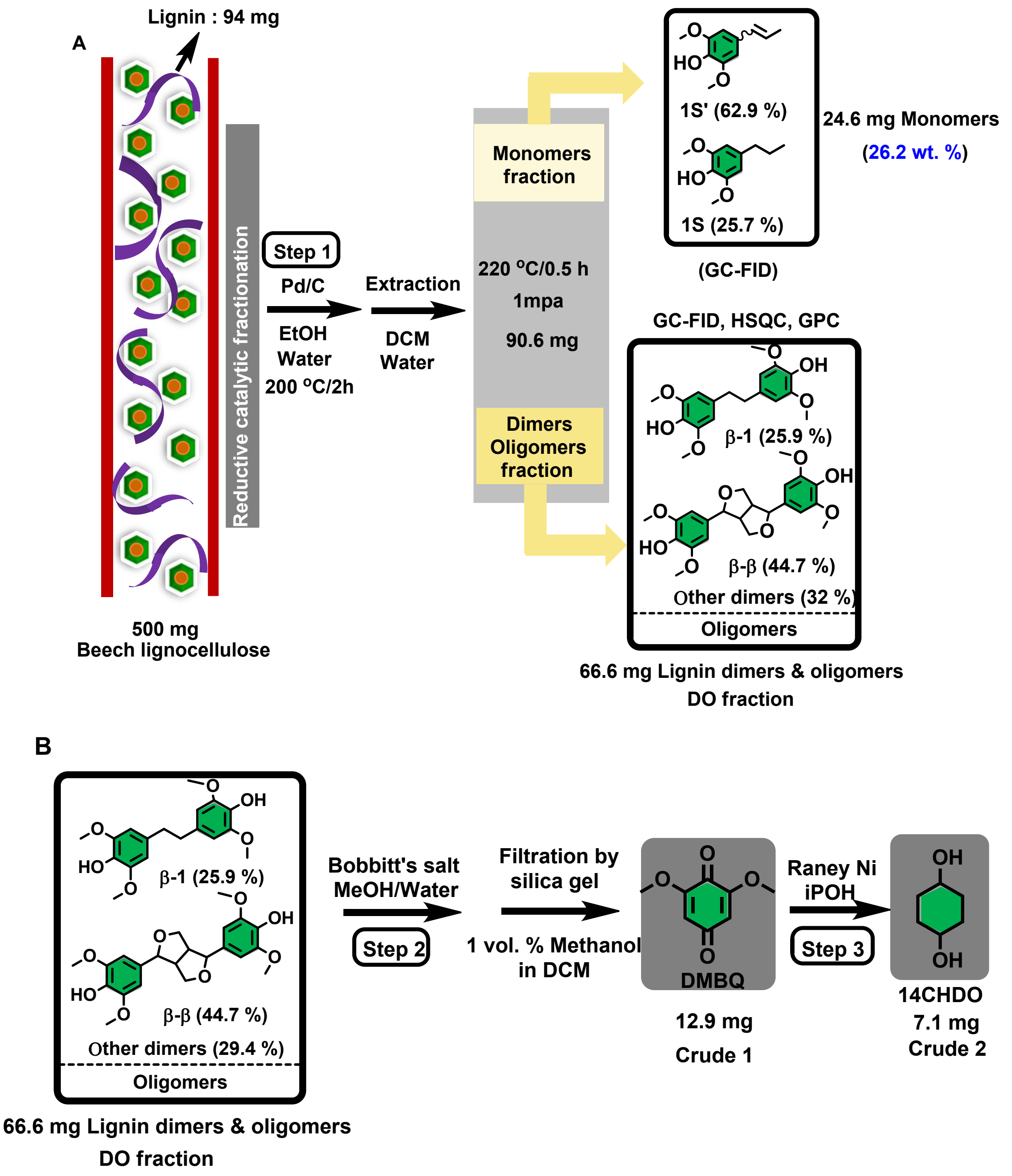

Figure S7. A) RCF of beech wood to lignin monomers and **DO** fraction; B) Catalytic conversion of **DO** fraction into **14CHDO**.

Step 1: The mild depolymerization of beech lignocellulose was carried out in a high-pressure Parr autoclave (50 mL), equipped with an overhead stirrer. Typically, the autoclave was charged with 50 mg of Pd/C catalyst, 500 mg of beech lignocellulose and ethanol (12 mL)/water (12 mL) as solvent. The reactor was sealed and flushed with N_2 at room temperature. Then, the reactor was heated to 200 °C and stirred at 400 rpm for 2 h. After completion of the reaction, the reactor was cooled to room temperature. Then 0.1 mL solution was collected through a syringe and injected to GC-MS or GC-FID after filtration through a PTFE filter (0.45 μm). The solid was separated from the solution by

centrifugation and subsequent decantation and additionally washed with ethanol (2×20 mL). The ethanol washings were combined in a round bottom flask and the solvent was removed *in vacuo*. **Extraction procedure:** To the obtained crude mixture, DCM (50 mL)/water (30 mL) was added and it was stirred at room temperature for 30 min. The suspension was then transferred into a 100 mL separating funnel and the DCM extracts were collected and combined in a round bottom flask. Then DCM extracts were dried over anhydrous $MgSO_4$. After filtration, the solvent was transferred to a round bottom flask and the solvent was removed in vacuo for further use as specified below. **Distillation procedure:** The DCM extracts were subjected to distillation at temperature 220 °C for 30 min using Kugelrohr apparatus under vacuum 1 mPa to give two fractions (low molecular-weight monomer fraction: 26.4 mg and high-molecular weight dimers and oligomers (namely **DO**), 66.6 mg. The monomer fraction was characterized by GC-FID/MS (**Figure S8**). The **DO fraction** was subsequently characterized by GC-FID/MS (**Figure S9**), GPC (**Figure S12),** HSQC (**Figure S14**). ***Step 2:*** The mild oxidative cleavage of **DO fraction** was carried out in a Schlenk reactor, equipped with a magnetic stirrer. Typically, a Schlenk reactor was charged with 66.6 mg of dimers and oligomers, 264 mg of Bobitt's salt, 7 mL methanol containing 0.4 wt% water relative to methanol mass. The reactor was sealed and flushed with Ar at room temperature. Then, the reactor was heated to 100 °C and stirred at 400 rpm for 0.5 h. After completion of the reaction, the reactor was cooled to room temperature. The crude mixture was subjected to purification by filtration via a short silica gel column using 1 vol. % methanol/DCM (200 mL) to get rid of all reduced bobitt's salt. The filtrates (**Crude 1**) was transferred in a round bottom flask and the solvent was removed in vacuo for further use as specified below. The obtained **Crude 1** was subsequently characterized by GC-FID/MS (**Figure S10**), GPC (**Figure S13),** HSQC (**Figure S15**). ***Step 3:*** The demethoxylation/hydrogenation of **Crude 1** was carried out in a 100 mL high-pressure Parr autoclave equipped with an overhead stirrer. Typically, the autoclave was charged with 200 mg Raney Ni catalyst, **Crude 1**, 15 mL isopropanol. The reactor was sealed and purged 3 times with H_2 and then pressurized with H_2 (30 bar). The reactor was then heated to temperature at 170 °C and stirred at 400 rpm for 10 h. After the reaction was completed, the reactor was cooled down to RT. The Raney Ni was separated from the solution by centrifugation and subsequent decantation and additionally washed with isopropanol (2×20 mL). The isopropanol washings were combined in a round bottom flask and the solvent was removed *in vacuo* to give **Crude 2,** which was subjected to further characterizations by GC-FID/MS (**Figure S11)** and HSQC (**Figure S16**).

2.5.1 GC-FID/MS traces of monomer and DO, Crude 1 and Crude 2 fraction

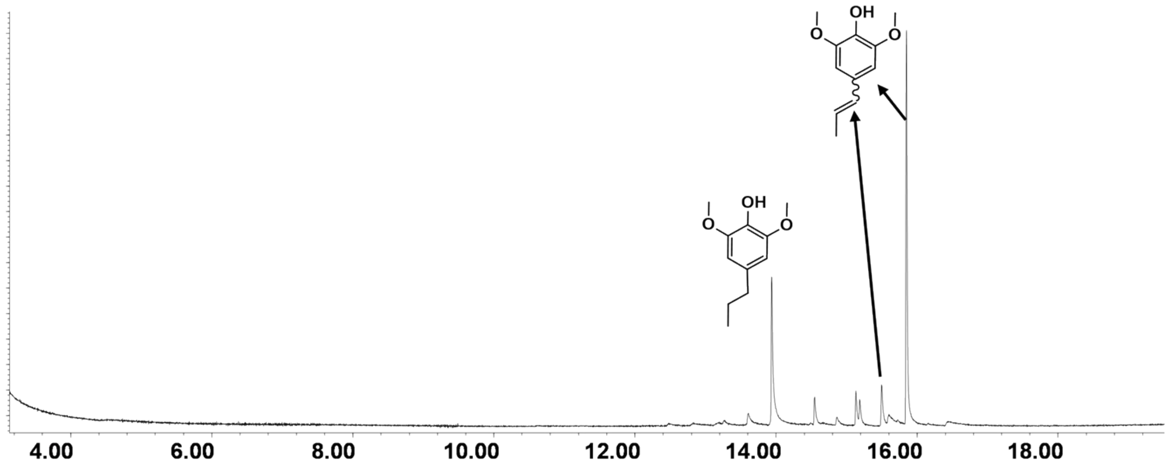

Figure S8. GC-FID trace of monomer fraction obtained from RCF of beech wood.

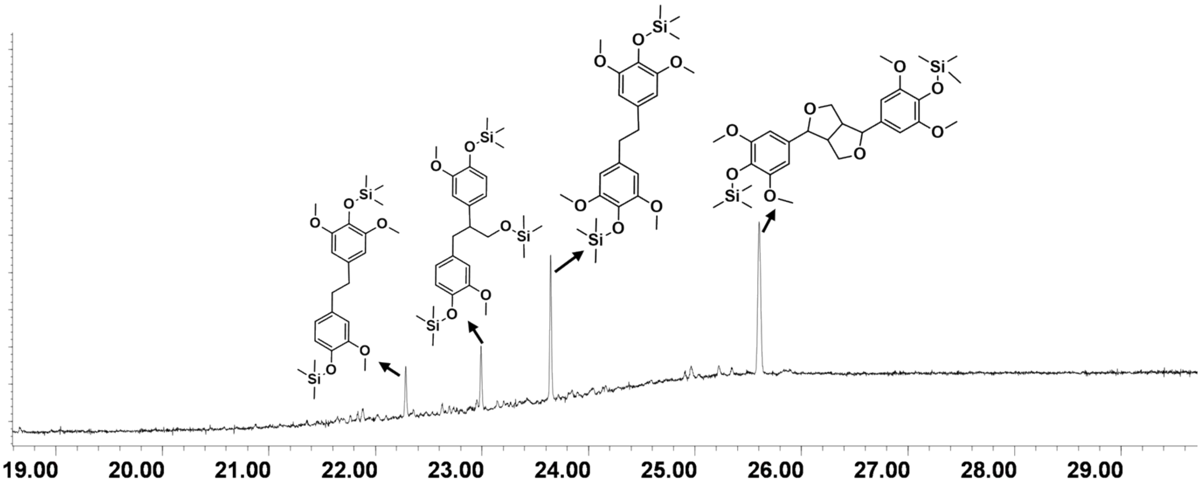

Figure S9. GC-FID trace of **DO** fraction obtained from RCF of beech wood.

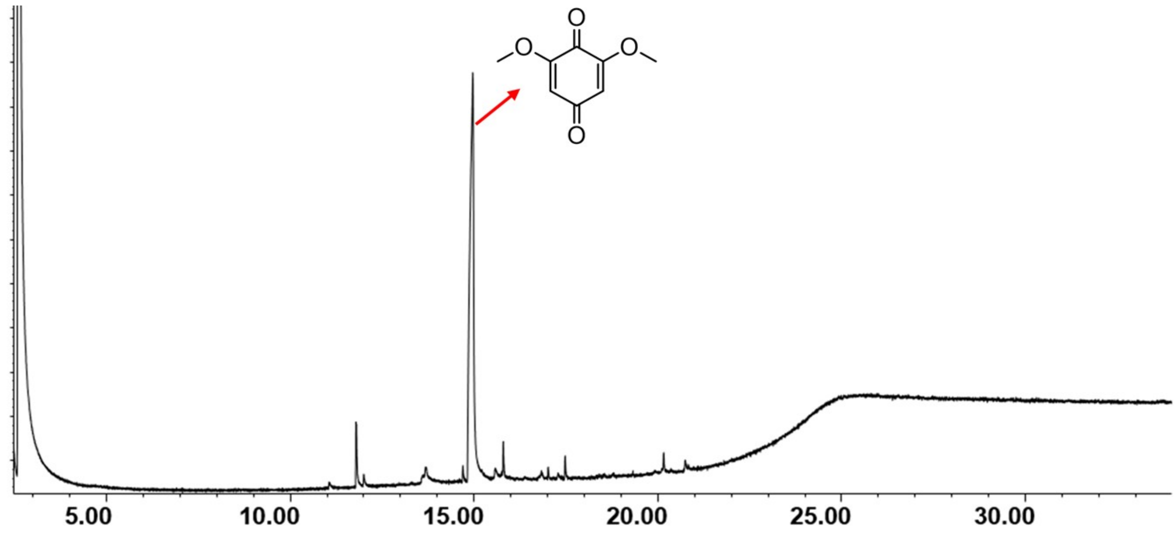

Figure S10. GC-FID trace of **Crude 1** obtained by the oxidative cleavage of **DO** over Bobitt's salt,

following by filtration via a short silica gel column chromatography using 1 vol % methanol/DCM.

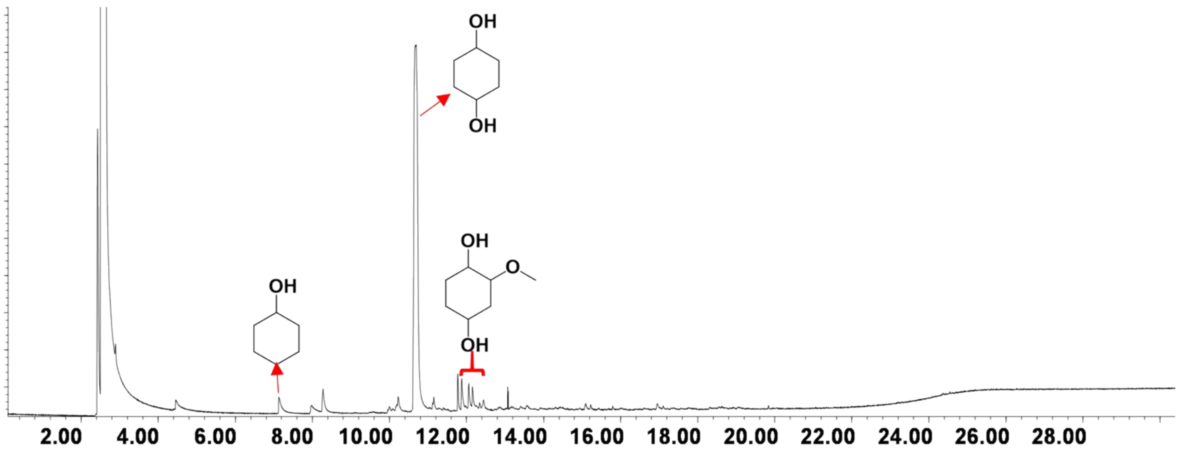

Figure S11. GC-FID trace of **Crude 2** obtained by catalytic demethoxylation and hydrogenation of **Crude 1** over Raney Ni catalyst.

2.5.2 GPC traces of monomer and DO and Crude 1 fraction

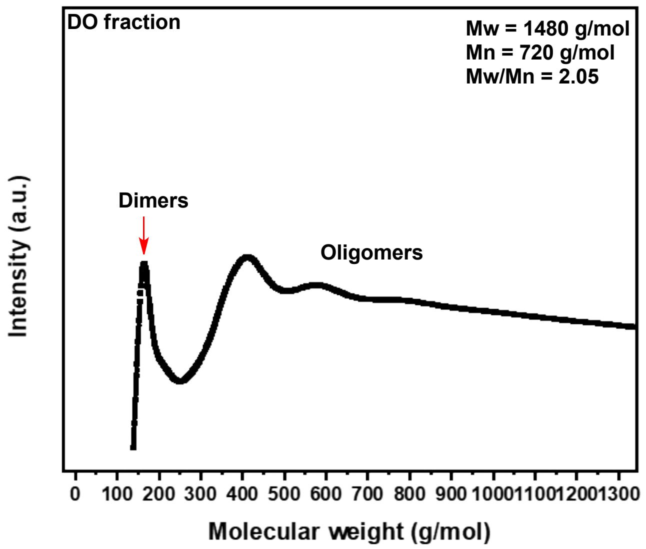

Figure S12. GPC trace of **DO** fraction.

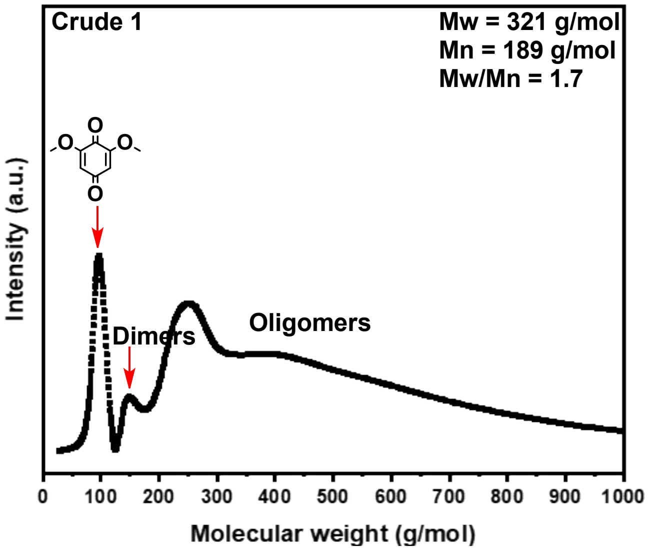

Figure S13. GPC trace of **Crude 1**.

2.5.3 2D HSQC spectra of DO, Crude 1 and Crude 2 fraction

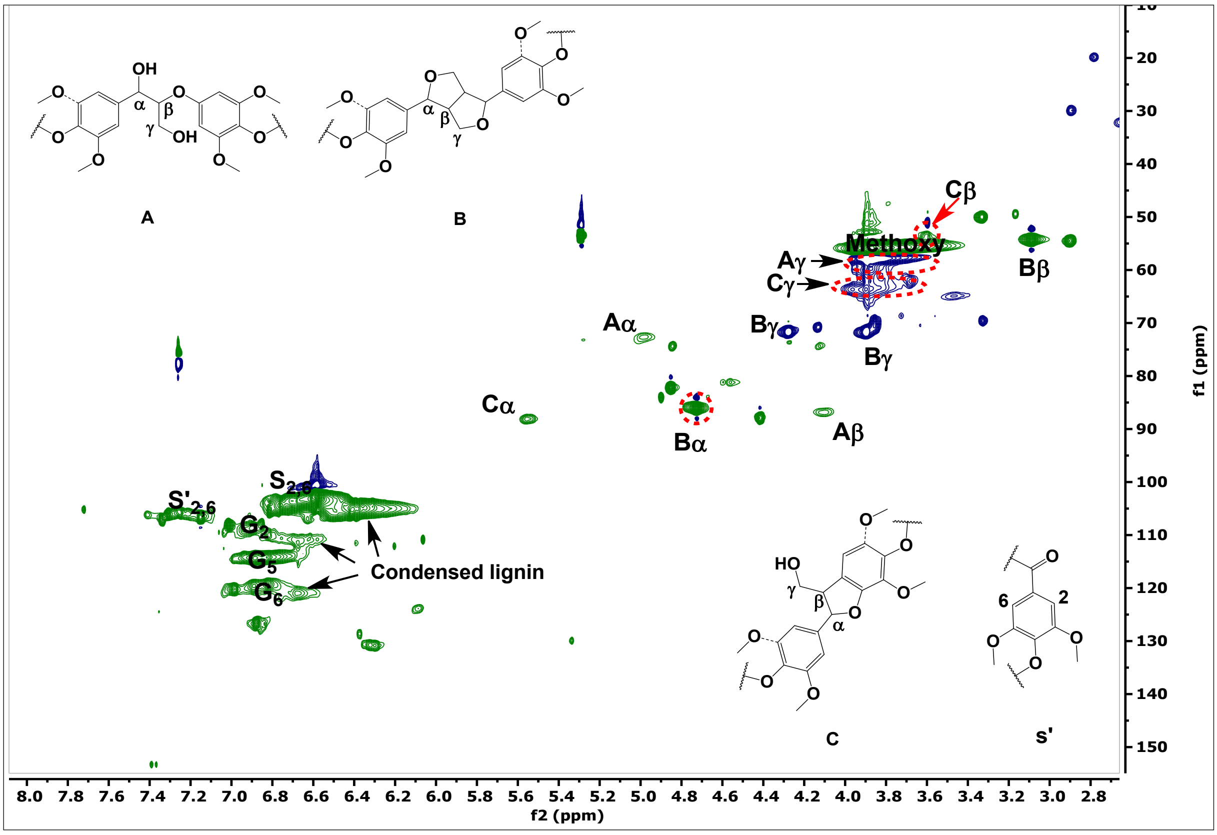

Figure S14. 2D HSQC spectrum of **DO** fraction.

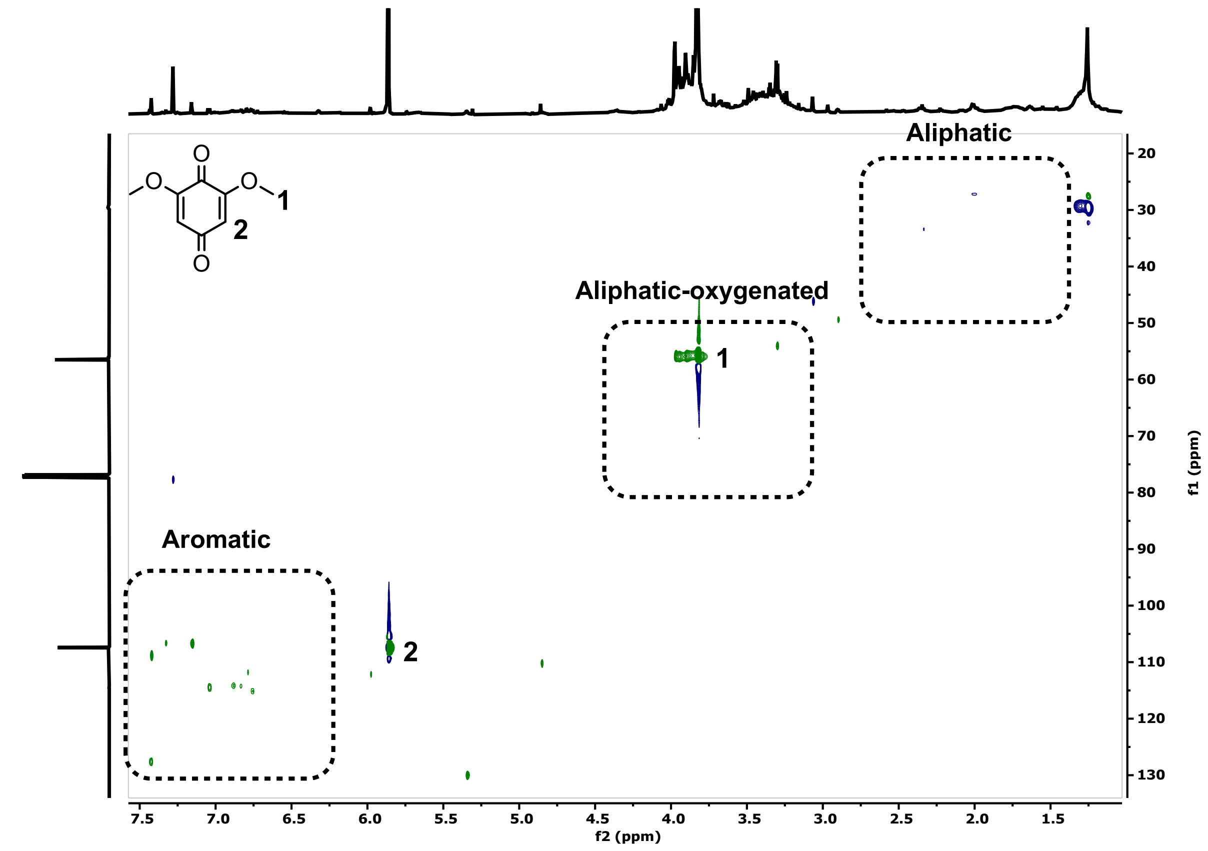

Figure S15. 2D HSQC spectrum of **Crude 1**

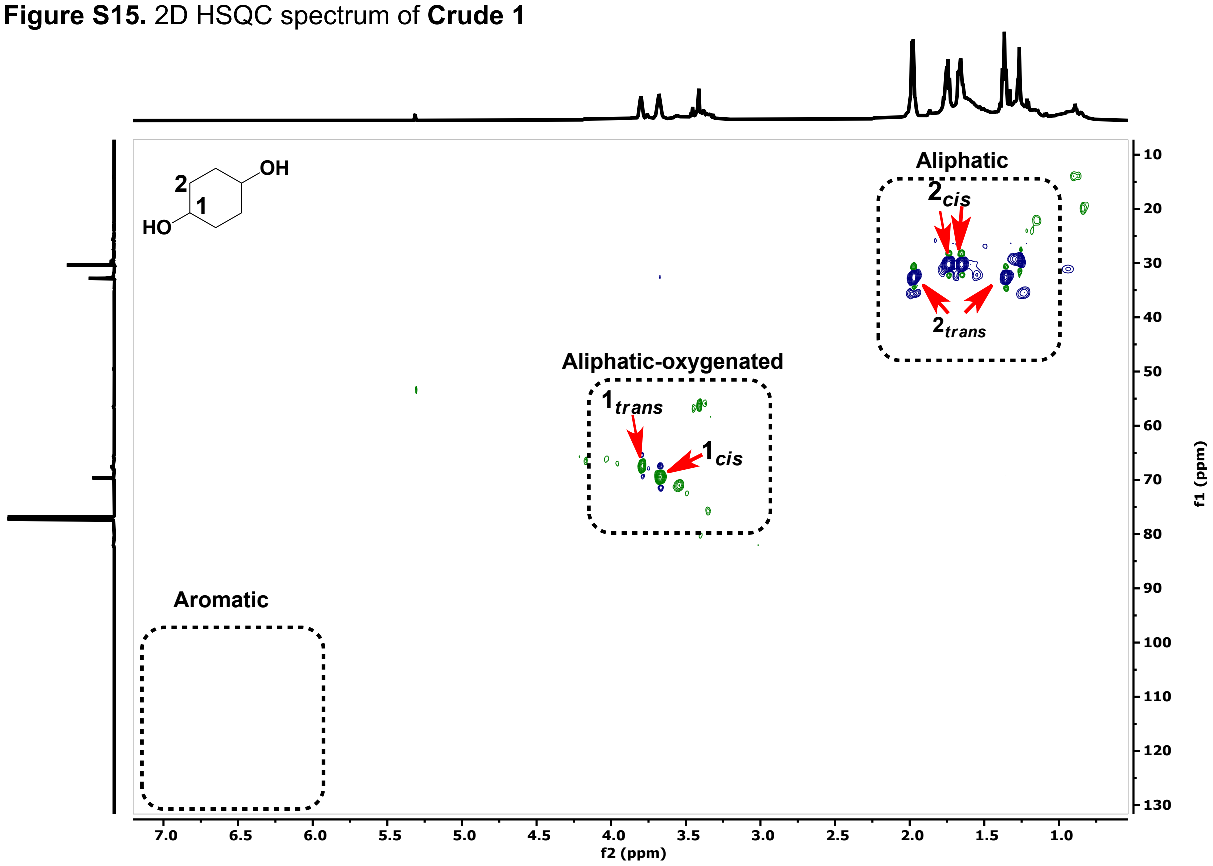

Figure S16. 2D HSQC spectrum of **Crude 2**

3. Catalytic direct amination of 14CHDO with ammonia to 14CHDA over Raney Ni catalyst

3.1 Detailed analysis of crude 14CHDA obtained from catalytic amination of 14CHDO

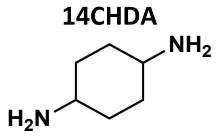

Reaction conditions: 0.5 mmol **14CHDO**, 100 mg Raney Ni catalyst, 2.5 mL *t*-amyl alcohol, 150 °C, 8 h, 5 mg dodecane as internal standard (for GC yield). After the reaction, the solvent *t*-amyl alcohol was removed under reduced pressure and liquid (49 mg) was obtained in an isolated yield of 86.0 %.

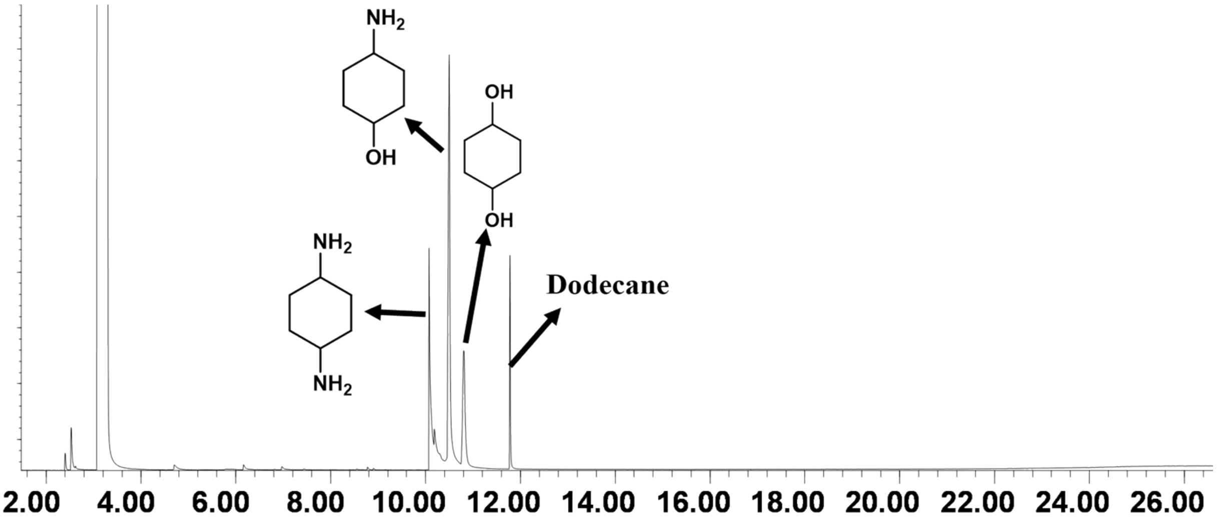

Figure S17. Catalytic direct amination of **14CHDO** with ammonia to **14CHDA** (0.5 mmol **14CHDO**, 100 mg Raney Ni catalyst, 2.5 mL *t*-amyl alcohol, 140 °C, 3 h, 5 mg dodecane)

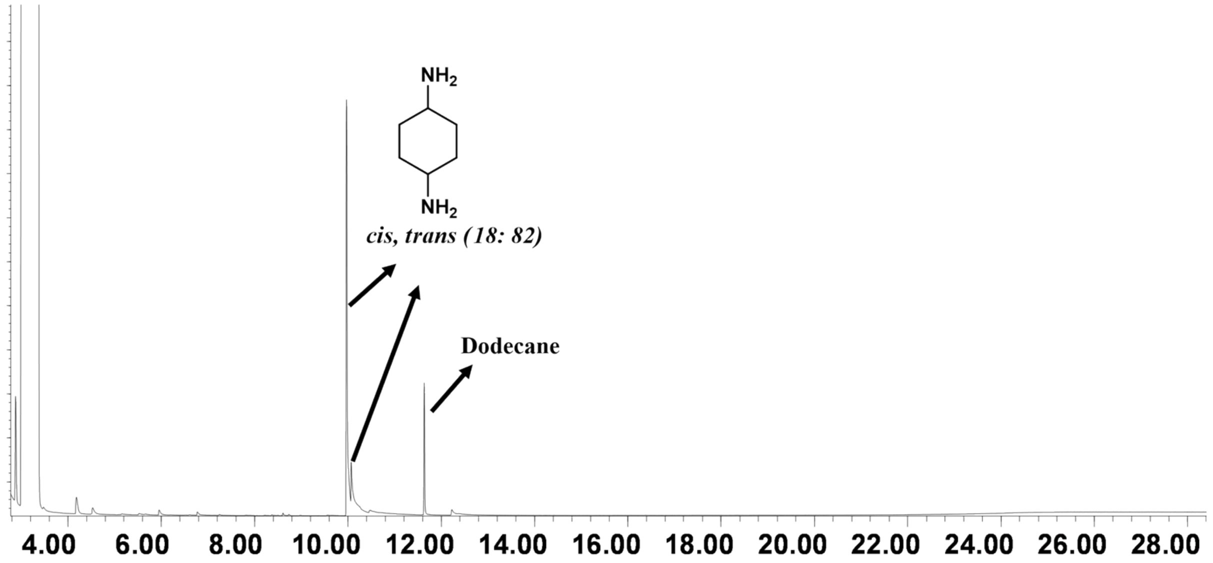

Figure S18. Catalytic direct amination of **14CHDO** with ammonia to **14CHDA** (0.5 mmol **14CHDO**, 100 mg Raney Ni catalyst, 2.5 mL *t*-amyl alcohol, 150 °C, 8 h, 5 mg dodecane)

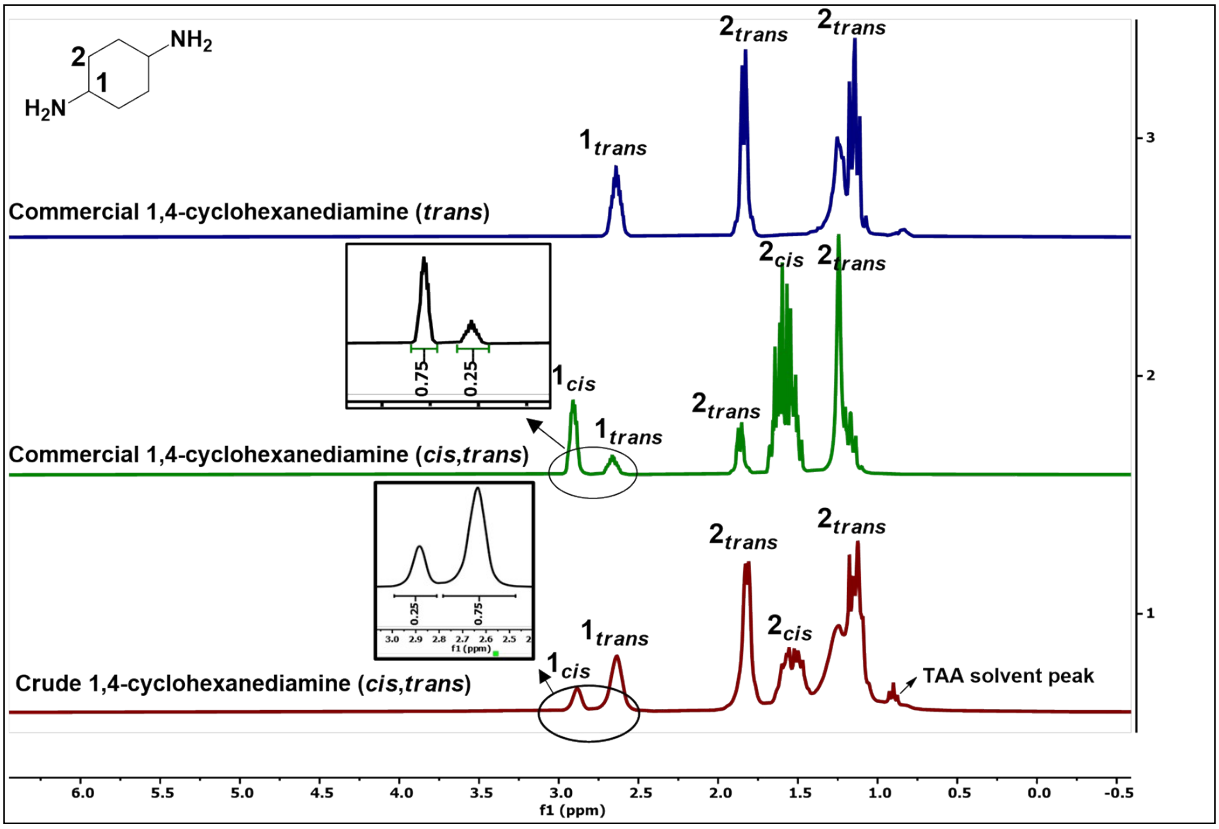

Figure S19. ^{1}H NMR of A) commercial **14CHDA** (trans); B) commercial **1,4CHDA** (cis and trans); C) isolated **14CHDA** (cis and trans) obtained from catalytic amination of **14CHDO** over Raney Ni**.**

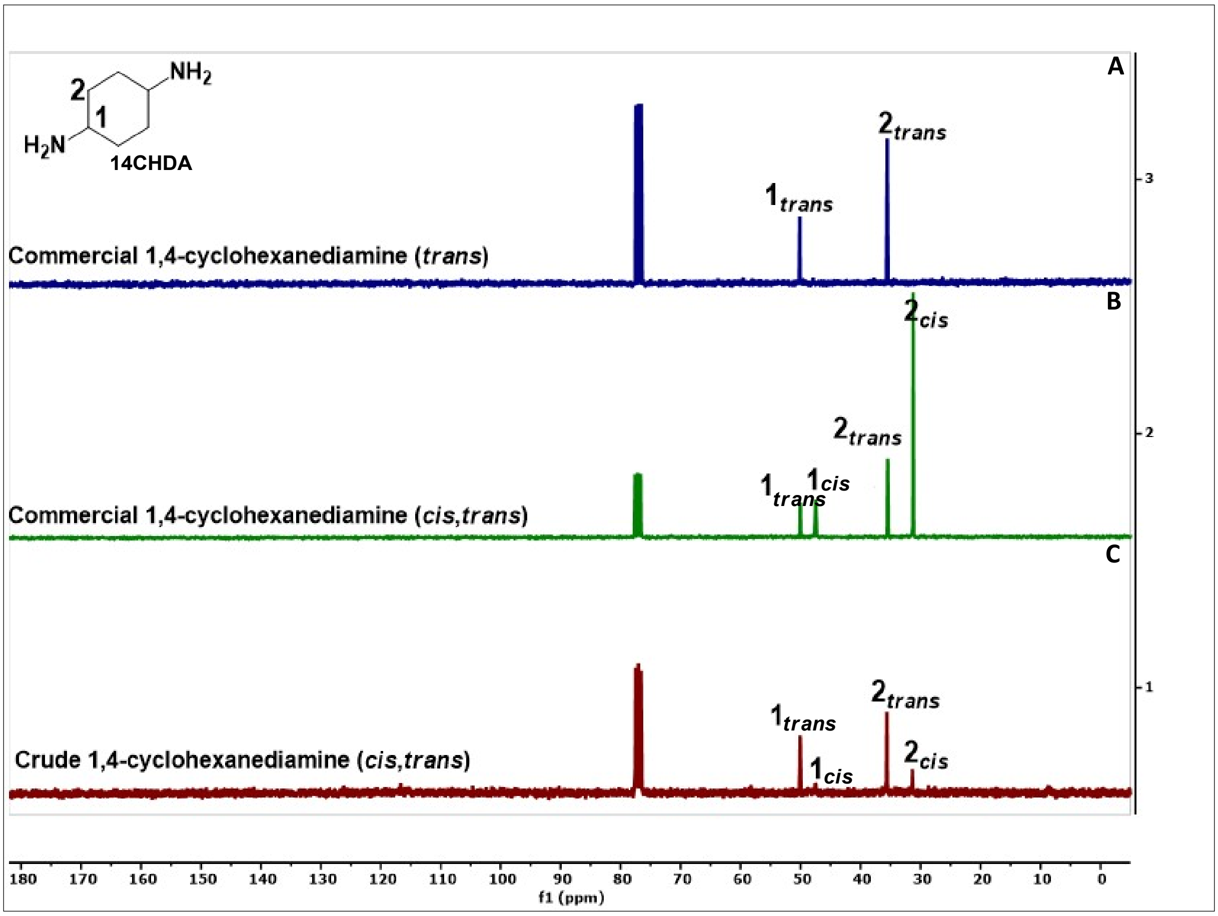

Figure S20. ^{13}C NMR of A) commercial **14CHDA** (trans); B) commercial **1,4CHDA** (cis and trans); C) isolated **14CHDA** (cis and trans) obtained from catalytic amination of **14CHDO** over Raney Ni**.**

3.2 Establishing the optimal reaction conditions for catalytic direct amination of 14CHDO to 14CHDA

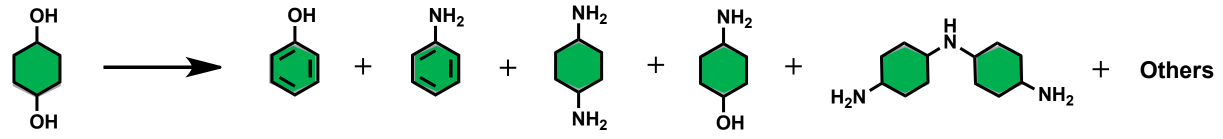

14CHDO **1B** **2B** **14CHDA** **3B** **4B**

Table S11. Survey of catalysts for direct amination of **14CHDO to 14CHDA** [a]

Catalyst	Conv. [b] (%)	Sel. (%) [b]					Yield (%) [c]
		**1B**	**2B**	**14CHDA**	**3B**	**4B**	
89 wt% Raney nickel	100	0	0	85.8	14.2	0	84.3
5 wt% Pd/C	10.0	14.5	85.5	0	0	0	0
5 wt% Pd/Al_2O_3	12.5	17.4	82.6	0	0	0	0
5 wt% Ru/C	19.6	0	0	0	54.6	45.4	0
5 wt%	0	0	0	0	0	0	0
65 wt% Ni/SiO_2	0	0	0	0	0	0	0
65 wt% Ni/SiO_2-Al_2O_3	0	0	0	0	0	0	0

[a]. Reaction conditions: **14CHDO** (0.5 mmol, 58 mg), 100 mg catalyst, 160 °C, 3 h, 2.5 mL *t*-amyl alcohol, 5 mg dodecane; Conversion and selectivity values were determined by GC-FID. Yield value determined by GC-FID using calibration curves and internal standard;

Table S12. Solvent Influence for catalytic direct amination of **14CHDO to 14CHDA** [a]

Solvent	Conv. (%)	Sel. (%) [b]					Yield (%) [c]
		**1B**	**2B**	**14CHDA**	**3B**	**4B**	
Methanol	0	0	0	0	0	0	0
H_2O	64.1	0	0	26.4	73.6	0	12.5
$NH_3 \cdot H_2O$[d]	18.3	0	0	0	66.1	33.9	0
2-MeTHF	100	0	0	96.1	3.9	0	95.7
Toluene	100	0	0	>99	0.1	0	98.4
TAA	100	0	0	>99	0.1	0	>99

[a]. Reaction conditions: **14CHDO** (0.5 mmol, 58 mg), 100 mg Raney Ni catalyst, 150 °C, 8 h, 2.5 mL solvents, 5 mg dodecane; [b]. Conversion and selectivity values were determined by GC-FID. [c]. Yield value determined by GC-FID using calibration curves and internal standard; [d]. 25 wt. % NH_3 in water.

Table S13. Influence of reaction temperature for catalytic direct amination of **14CHDO to 14CHDA** [a]

Temperature (°C)	Conv. [b] (%)	Sel. (%) [b]					Yield (%) [c]
		**1B**	**2B**	**14CHDA**	**3B**	**4B**	
120	24.2	0	0	4.8	95.2	0	0.9
130	57.0	0	0	15.9	84.1	0	13.6
140	79.3	0	0	41.2	58.8	0	34.0
150	89.1	0	0	58.9	41.1	0	49.4
160	100	0	0	85.2	14.8	0	84.4
170	100	0	0	>99	0.6	0	>99

[a]. Reaction conditions: **14CHDO** (0.5 mmol, 58 mg), 100 mg Raney Ni catalyst, 120-170 °C, 3 h, 2.5 mL *t*-amyl alcohol, 5 mg dodecane; [b]. Conversion and selectivity values were determined by GC-FID; [c]. Yield value determined by GC-FID using calibration curves and internal standard;

Table S14. Influence of reaction time for catalytic direct amination of **14CHDO to 14CHDA** [a]

Time (h)	Conv.[b] (%)	Sel. (%) [b]					Yield (%) [c]
		**1B**	**2B**	**14CHDA**	**3B**	**4B**	
0.5	49.7	0	0	11.6	88.4	0	9.1
1.5	84.3	0	0	32.3	67.7	0	19.8
2.5	93.0	0	0	58.9	41.1	0	45.9
4	96.6	0	0	76.2	23.8	0	62.6
6	100	0	0	88.2	11.8	0	76.6
8	100	0	0	>99	0.4	0	>99
10	100	0	0	>99	0	0	>99

[a]. Reaction conditions: **14CHDO** (0.5 mmol, 58 mg), 100 mg Raney Ni catalyst, 150 °C, 3 h, 2.5 mL *t*-amyl alcohol, 5 mg dodecane; [b]. Conversion and selectivity values were determined by GC-FID; [c]. Yield value determined by GC-FID using calibration curves and internal standard;

4. Reference

1. E. Subbotina, T. Rukkijakan, M. D. Marquez-Medina, X. W. Yu, M. Johnsson and J. S. M. Samec, *Nat. Chem.*, 2021, **13**, 1118-1125.
2. N. Q. Ran, D. R. Knop, K. M. Draths and J. W. Frost, *J Am Chem Soc*, 2001, **123**, 10927-10934.
3. C. F. Huang, Y. H. Chou, Process for producing hydroquinone and derivates, US9630899B1, 2017.
4. A. Hassan, E. Bagherzadeh, R. G. Anthony, G. G. Borsinger, A. Hassan, System and process for production of aniline and toluenediamine. *U.S. Patent No. 8,153,076*. 2012, Washington, DC: U.S. Patent and Trademark Office.
5. R. T. Driessen, P. Kamphuis, L. Mathijssen, R. Zhang, L. G. J. van der Ham, H. van den Berg and A. J. Zeeuw, *Chem. Eng. Technol*, 2017, **40**, 838-846.
6. H. J. Wang, L. Zhang, B. B. Li, D. Wang, X. Q. Wang, Method for synthesizing 1,4-cyclohexanediol through catalytic hydrogenation of hydroquinone, CN101811936A, 2010.
7. G. Y. Bai, F. Li, X. X. Fan, Y. L. Wang, M. D. Qiu, Z. Ma and L. B. Niu, *Catal Commun*, 2012, **17**, 126-130.
8. Z. G. Qiu, C. S. Yong, Method for preparing 1,4-cyclohexanediamine at high pressure. CN103896778A, 2014.
9. A. Fischer, T. Mallat and A. Baiker, *J. Catal.,* 1999, **182**, 289-291.
10. H. X. Ma and J. G. Cai, *Russ. J. Appl. Chem.*, 2014, **87**, 397-403.
11. Y. J. Ding, T. Wang, Y. Lv, L. X. Ma, Method for preparing cyclohexanediamine through catalytic hydrogenation, CN102690204B, 2014.
12. T. A. Weil, Process for 1,4-phenylenediamine, US4400536A, 1983.
13. X. W. Wang, Method for preparing p-phenylenediamine, CN104292109A, 2015
14. X. Y. Wang and R. Rinaldi, *Energ Environ Sci*, 2012, **5**, 8244-8260

15. X. Y. Wu, M. V. Galkin, K. Barta, Chem. Cat., 2021, 1, 1-14.
16. X. Y. Wu, M. De Bruyn and K. Barta, *ChemSusChem*, 2022, **15**, e202200914.
17. Z. H. Sun, Z. H. Zhang, T. Q. Yuan, X. H. Ren and Z. M. Rong, *ACS Catal.*, 2021, **11**, 10508-10536.

Supplementary Note 1: Proposed industrial pathways for 14CHDO and 14CHDA production

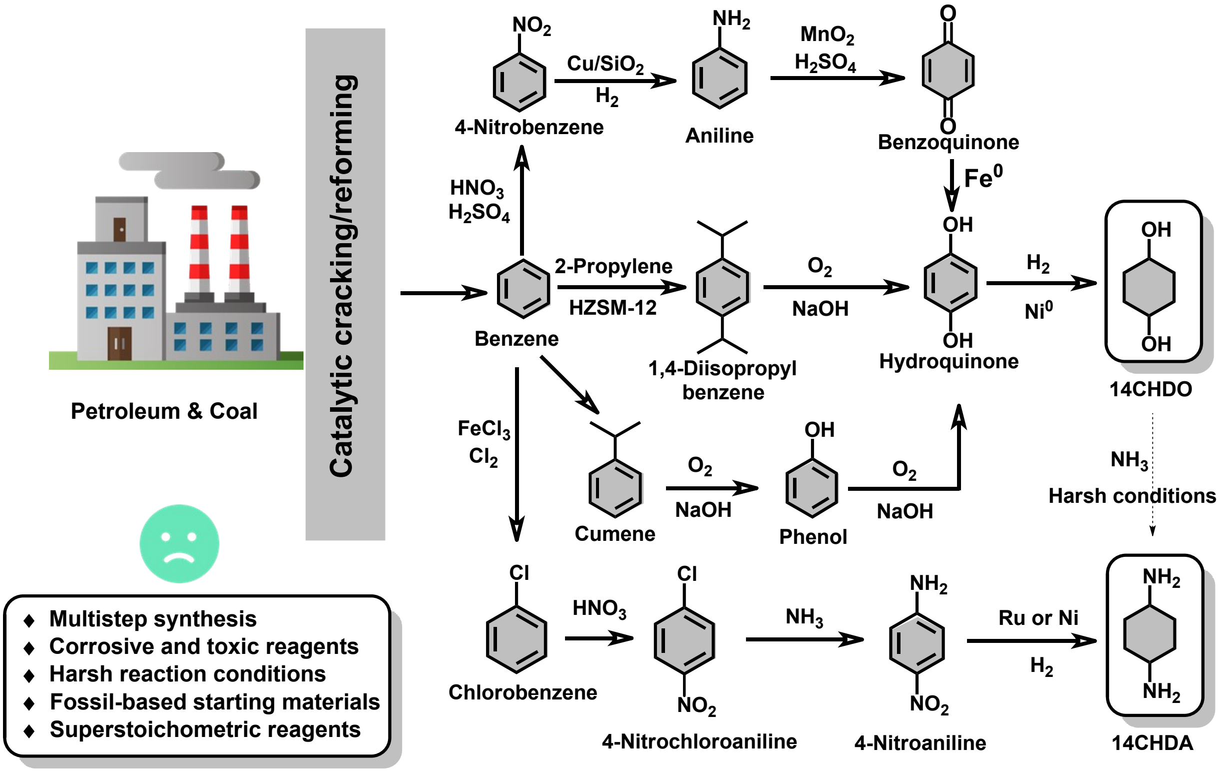

Figure S21: Proposed industrial pathways for 14CHDO and 14CHDA production

Central to the synthesis of 14CHDO is the formation of hydroquinone (HQ). Three reaction pathways to HQ are known: a) aniline oxidation in the presence of MnO_2 and H_2SO_4;[2] b) oxidative cleavage of diisopropylbenzene and c) phenol hydroxylation.[3] While the former route links in with the well-established, yet hazardous, synthesis of aniline via benzene/nitrobenzene, [3-5] the latter two routes centre by multi-step Hock-type processes with the simultaneous production of sizeable amounts of useful acetone. Finally, 14CHDO can be derivable by direct hydrogenation of HQ.[6-7] 14CHDA can be prepared from 14CHDO by contacting the latter compound with liquid ammonia or supercritical ammonia in a high-pressure reactor at elevated temperatures (200-250 °C).[8-9] However, the more common methodology to 14CHDA is by hydrogenation of 1,4-phenylenediamine (14PDA) in the presence of Ru catalysts.[10-11] The most common method to prepare 14PDA is via the reduction of p-nitroaniline (pNA). The main drawbacks to this process revolve around the nitration step, most usually on chlorobenzene, and the separation of ortho/para isomers.[12] An alternative route starts with reacting urea with nitrobenzene in the presence of base, yielding 4-nitrosoaniline and 4-nitroaniline, followed by

a catalytic hydrogenation step. This process requires the use of a large amount of urea as to avoid the formation of 4,4'-dinitrophenylamine.[13]

Supplementary Note 2: Considerations for the high catalytic performance of Raney Nickel catalyst for catalytic demethoxlation and hydrogenation of DMBQ

Raney Ni was tested in this case as it has previously already shown great reactivity for selective demethoxylation and reduction of phenolic rings in an excellent work of Rinaldi[14] and in our previously published work.[15,16] It is also to be noted that the other metal supported catalysts that were tested in this work possess a higher affinity for aromatic ring reduction compared to Raney Nickel. As a result, facile demethoxylation over aromatic ring hydrogenation is favored. This can also be confirmed by the fact that the reaction rate constant for demethoxylation starting from the saturated ring is much slower than unsaturated aromatic ring.

Furthermore, Raney nickel catalyst is also a highly active transfer hydrogenation catalyst when using isopropanol as solvent.[14] The facile hydrogen abstraction from the H-donor isopropanol can also facilitate the demethoxylation and hydrogenation of DMBQ.

In addition, we also speculate that the high catalytic efficiency of the Raney Nickel catalyst in demethoxylation and hydrogenation of DMBQ is favored by the intimate Ni-Al interaction in Raney Nickel[17] as opposed to other metal supported catalysts that were tested. However, this still needs to be proved in future work.
